# Genomic epidemiology of *Mycobacterium bovis* infection in sympatric badger and cattle populations in Northern Ireland

**DOI:** 10.1099/mgen.0.001023

**Published:** 2023-05-25

**Authors:** Assel Akhmetova, Jimena Guerrero, Paul McAdam, Liliana C. M. Salvador, Joseph Crispell, John Lavery, Eleanor Presho, Rowland R. Kao, Roman Biek, Fraser Menzies, Nigel Trimble, Roland Harwood, P. Theo Pepler, Katarina Oravcova, Jordon Graham, Robin Skuce, Louis du Plessis, Suzan Thompson, Lorraine Wright, Andrew W. Byrne, Adrian R. Allen

**Affiliations:** ^1^​ University of Glasgow, Glasgow, UK; ^2^​ Hospital Juarez de Mexico, Mexico City, Mexico; ^3^​ Fios Genomics, Edinburgh, UK; ^4^​ Department of Infectious Diseases, College of Veterinary Medicine, University of Georgia, Athens, GA, USA; ^5^​ Institute of Bioinformatics, University of Georgia, Athens, GA, USA; ^6^​ Center for the Ecology of Infectious Diseases, University of Georgia, Athens, GA, USA; ^7^​ Foreign Commonwealth and Development Office, Glasgow, UK; ^8^​ Department for the Economy, Belfast, UK; ^9^​ Agrifood and Biosciences Institute, Belfast, UK; ^10^​ University of Edinburgh, Roslin Institute, Edinburgh, UK; ^11^​ Department of Agriculture, Environment and Rural Affairs (DAERA), Belfast, UK; ^12^​ Farmvet Systems Ltd, Moneymore, UK; ^13^​ Department of Biosystems Science and Engineering, ETH Zurich, Basel, Switzerland; ^14^​ Swiss Institute of Bioinformatics, Lausanne, Switzerland; ^15^​ Department of Agriculture Food and the Marine (DAFM), Dublin, Ireland; ^†^​ Present address: School of Animal and Comparative Biomedical Sciences, University of Arizona, AZ, Tucson, USA

**Keywords:** bovine tuberculosis, genome sequencing, transmission dynamics

## Abstract

Bovine tuberculosis (bTB) is a costly, epidemiologically complex, multi-host, endemic disease. Lack of understanding of transmission dynamics may undermine eradication efforts. Pathogen whole-genome sequencing improves epidemiological inferences, providing a means to determine the relative importance of inter- and intra-species host transmission for disease persistence. We sequenced an exceptional data set of 619 *Mycobacterium bovis* isolates from badgers and cattle in a 100 km^2^ bTB ‘hotspot’ in Northern Ireland. Historical molecular subtyping data permitted the targeting of an endemic pathogen lineage, whose long-term persistence provided a unique opportunity to study disease transmission dynamics in unparalleled detail. Additionally, to assess whether badger population genetic structure was associated with the spatial distribution of pathogen genetic diversity, we microsatellite genotyped hair samples from 769 badgers trapped in this area. Birth death models and TransPhylo analyses indicated that cattle were likely driving the local epidemic, with transmission from cattle to badgers being more common than badger to cattle. Furthermore, the presence of significant badger population genetic structure in the landscape was not associated with the spatial distribution of *M. bovis* genetic diversity, suggesting that badger-to-badger transmission is not playing a major role in transmission dynamics. Our data were consistent with badgers playing a smaller role in transmission of *M. bovis* infection in this study site, compared to cattle. We hypothesize, however, that this minor role may still be important for persistence. Comparison to other areas suggests that *M. bovis* transmission dynamics are likely to be context dependent, with the role of wildlife being difficult to generalize.

## Data Summary

All sequence data have been deposited in the National Center for Biotechnology Information’s (NCBI’s) Short Read Archive (SRA) and is publicly available – BioProject PRJNA925930 (https://www.ncbi.nlm.nih.gov/bioproject/PRJNA925930). Badger genetic data were previously used in an analysis on badger relatedness and movement ecology from the TVR study zone [73] and the data are open access and free to use in this study.

Supplementary data, R scripts and beast xml run files used in the performance of this work are curated at the following GitHub repository: https://github.com/AdrianAllen1977/Genome-epidemiology-of-Mycobacterium-bovis-infection-in-contemporaneous-sympatric-badger-and-cattle.

Locations of cattle herds have been removed from Data S1 (available in the online version of this article) to protect personal data. Locations of badgers are still recorded.

**Impact Statement**

Bovine tuberculosis (bTB) is a major burden on the livestock industries of the UK and Ireland. The European badger (*Meles meles*) has been implicated as a potential wildlife maintenance host and impediment to disease eradication. Previous molecular epidemiological investigations have shown that cattle and badgers share similar strains of the causative organism, *Mycobacterium bovis *, suggestive of ongoing interspecies transmission at a broad geographical scale, but until recently assessing the relative importance of intra- and interspecies transmission has been difficult. Our study adds to the growing body of evidence that uses bacterial whole-genomic data to inform on the transmission dynamics in this disease epidemiological system (epi-system). We found that in a small area in Northern Ireland that experienced elevated disease incidence, cattle were largely driving infection dynamics. We contrast this with other areas in the UK, which observed greater badger involvement, noting that context-specific, spatio-temporal variation in disease dynamics is a probable feature of the wider bTB problem.

## Introduction

*Mycobacterium bovis* infection, causing bovine tuberculosis (bTB) in cattle (*Bos taurus*) and badgers (*Meles meles*), is a persistent and costly problem for the farming industries and governments of the UK and Ireland [1]. In Northern Ireland the bTB eradication scheme cost UK £44 million in 2017/2018 [2]. The complex epidemiology of the disease epidemiological system (epi-system) is well recognized, with the role of wildlife in transmitting infection to cattle acknowledged as an impediment to eradication [3]. A major knowledge gap for this disease has been, until recently, a detailed understanding of inter-host transmission dynamics and their relative importance [4].

Multi-host zoonotic infections of slowly evolving pathogens, such as the members of the *Mycobacterium tuberculosis* complex (MTBC), present significant challenges to researchers who wish to use molecular methods to understand disease transmission [5]. Previously, multi-locus variable number of tandem repeats analysis (MLVA) and spoligotyping were used to characterize spatio-temporal patterns in bTB epidemiology [6–9]. These methods have demonstrated how *M. bovis* infections present as a series of geographically localized micro-epidemics [7, 9]. However, MLVA and spoligotype loci, whilst useful in defining the home ranges of endemic infections [8, 10], evolve at slower rates than the inter-host transmission rate, thereby limiting their utility for contemporary outbreak investigations [11].

Whole-genome sequencing (WGS) technologies and associated phylogenetic analytical frameworks have helped to reveal sources of infection and improve surveillance and control for various pathogens [12–14]. These phylodynamic methods have been applied most effectively to fast-evolving viral pathogens, whose mutation rates can, with dense sampling, permit inference of disease dynamics, over short time intervals [5, 15]. While the latter degree of resolution may be unobtainable for slowly evolving bacterial pathogens, recently it has been shown that, provided dense sampling is undertaken across a wide temporal window, much can be revealed about the inter-host disease transmission dynamics of slowly evolving pathogens, such as *M. bovis* [16–22].

Pathogen spread across landscapes is recognized to be an inherently spatial process [23], leading to distinct patterns in pathogen genetic structure. Similarly, free-ranging wildlife hosts exhibit partitioning of their own genetic variation across landscapes [24], for example, isolation by distance (IBD) [25]. An appreciation of how these types of landscape–genetic phenomena intersect can help to inform epidemiological investigations in wildlife populations [23]. A key question within localized bTB micro-epidemics is whether significant badger population structure is observed over small geographical scales, and if so, whether it explains any of the partitioning of *M. bovis* genetic variation in the landscape.

**Fig. 1.**
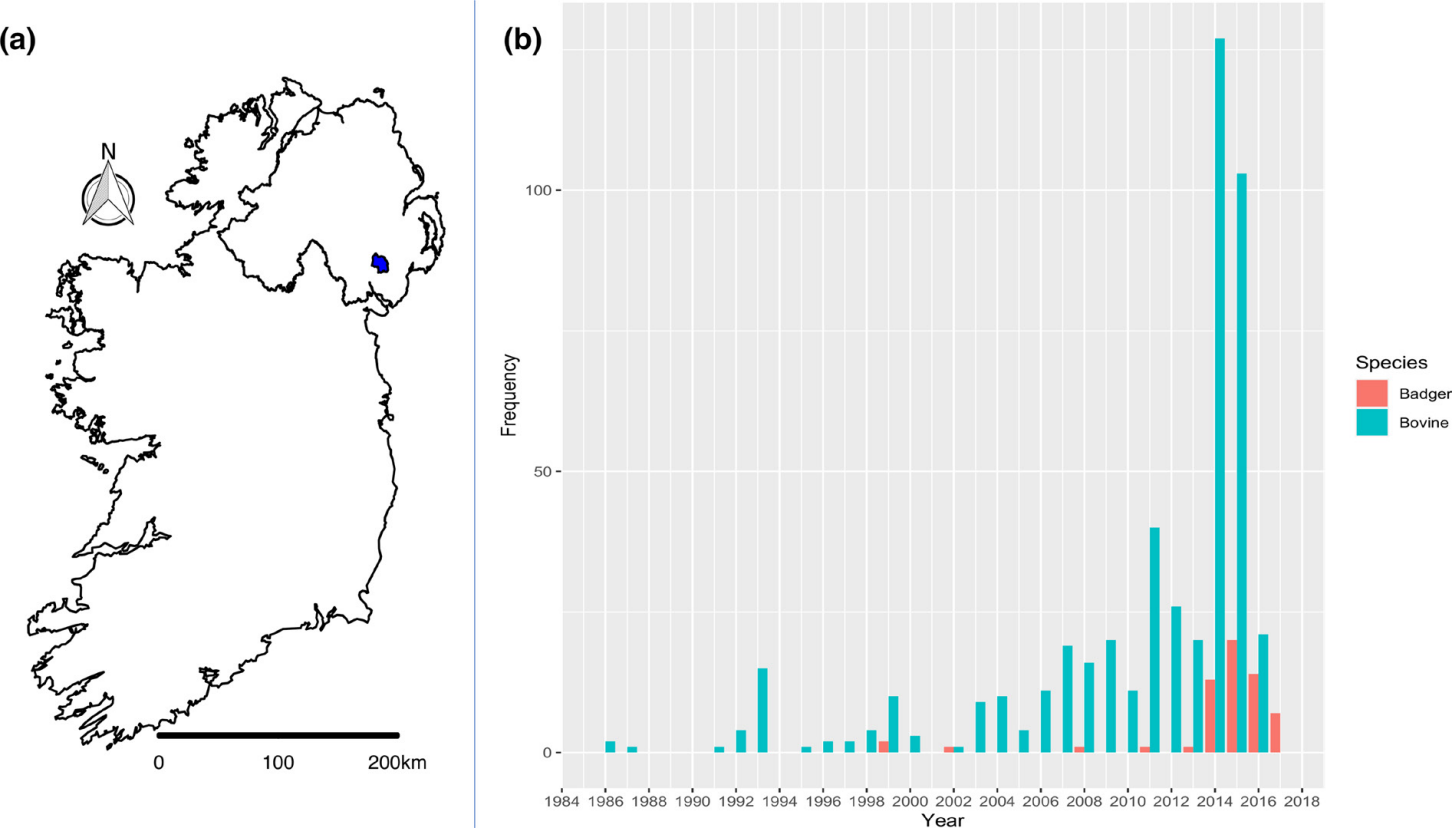
(a) Map of the island of Ireland with the location of the 100 km^2^ TVR cull zone highlighted in red. (b) Frequency bar plot of *Mycobacterium bovis* isolates sampled annually, from unique badgers and cattle, in the TVR zone prior to (1986–2013) and during (2014–2017) the intervention.

In this study we sought to improve understanding of the interspecies transmission dynamics of *M. bovis* and the effect of badger population structure on the spatial partitioning of pathogen diversity. We undertook a systematic sampling of both cattle and badgers in a small 100 km^2^ area of Northern Ireland (Fig. 1a), using samples and data from a recently completed (2014–2018) wildlife intervention – The ‘test and vaccinate or remove’ (TVR) selective badger culling protocol [26, 27]. This data set provided an opportunity to apply WGS, phylodynamic and population genetic methods to determine the roles that both cattle and badgers play in the local disease dynamics.

## Methods

### Sampling of cattle and badgers

The TVR zone (Fig. 1a) was chosen because it has a high incidence of cattle TB [24 % confirmed bTB incidence over 2 years (2011/2012)] and is embedded within County Down, which also has one of the highest average badger densities (3.88 badgers km^−2^) in Northern Ireland [28].

An initial survey was conducted to establish sett locations [29]. Overnight cage trapping was used across all years between the months of May and October 2014–2018. Trapped badgers were anaesthetized, trap-side, hair sampled and tested for *M. bovis* infection using the dual-path platform (DPP) serology test based on the StatPak method [30]. Tracheal aspirate was taken from all trapped badgers, whether DPP-positive or -negative and sent for bacteriological culture as described below. From 2015–2018, DPP-positive badgers were humanely euthanized and DPP-negative badgers were vaccinated using injectable bacillus Calmette–Guérin (BCG) and released [27]. Culled badgers underwent post-mortem examination according to a standardized protocol [31] and specified tissues were submitted for bacteriological culture.

All cattle in Northern Ireland are TB tested at least annually using the standardized [32] single intradermal comparable cervical tuberculin (SICCT) test [33]. Specified tissue from all SICCT-positive bTB reactor cattle in the TVR region from 2014 to 2017 were harvested at the time of slaughter and submitted for bacteriological culture.

To provide extra temporal depth, a selection of historical isolates (*n*=243) from both hosts in the TVR zone were recultured from the Agrifood and Biosciences Institute (AFBI) *M. bovis* Northern Ireland-wide strain archive. The temporal window for all isolates runs from 1986 to 2017. All historical badger isolates were derived from road traffic accident (RTA) post-mortems in a surveillance scheme run by the Department of Agriculture, Environment and Rural Affairs in Northern Ireland (DAERA-NI) [34].

### *M. bovis* culture and genomic DNA extraction

*M. bovis* isolates were initially cultured in the liquid BD BACTEC MGIT 960 system, solid Stonebrinks and Löwenstein–Jensen media and single colonies were selected for sub-culture [35]. Isolates were heat-killed in a water bath at 80 °C for 30 min. DNA was extracted using standard high-salt/cationic detergent cetyl hexadeycl trimethyl ammonium bromide (CTAB) and solvent extraction protocols [36, 37].

### Spoligotyping and MLVA analysis

*M. bovis* isolates were genotyped by spoligotyping [6] and eight-loci MLVA using previously described methods [7]. Authoritative names for spoligotype patterns were obtained from www.mbovis.org [38]. MLVA profiles were named using a laboratory nomenclature [35].

### Genome sequencing and bioinformatic analyses

Sequencing libraries were prepared using the Illumina Nextera XT method to produce inserts of approximately 500–600 bp. One hundred samples were sequenced at AFBI using an Illumina MiSeq platform with Illumina V2 chemistry, producing paired-end reads of 250 bp. A further 100 samples were sequenced at the Glasgow Polyomics facility using an Illumina Miseq producing 2×300 bp paired end reads. All remaining samples were sequenced by Eurofins Scientific using an Illumina HiSeq producing 2×250 bp paired end reads.

Reads for each sample were mapped to the recently updated/annotated [39] reference genome for *M. bovis* strain AF2122/97 (GenBank accession LT708304.1) using the mapping-based phylogenomics pipeline, RedDog V1beta.10.3 [40], to identify single-nucleotide polymorphism (SNP) variants across all isolates. Alignment and mapping were carried out in Bowtie 2 v2.2.9 [41] using the sensitive local mapping setting. SNP calling was undertaken using SAMtools and BCFtools [42] using the consensus caller setting. Using the RedDog configuration file to set desired cutoffs, we opted for a minimum depth of 10× for SNP calling. The average coverage failure filter, average depth filter and average mapping failure filters were set at 95 %, 10× and 70 %, respectively. Transposable elements, repeat regions and the PE/PPE regions as defined in the GenBank annotation were excluded from SNP calling using the parseSNP tool in the RedDog pipeline [40].

The final RedDog output is a whole-genome alignment composed of SNPs compared to the reference sequence.

Some isolates were sequenced by Glasgow Polyomics and others were sequenced by Eurofins laboratories. Fifteen randomly selected isolates were resequenced by AFBI.

### Badger microsatellite genotyping

Nuclear DNA extracted from all badger hair samples collected 2014–2018 was genotyped at 14 microsatellite loci using previously described methods [24].

### Testing for isolation by distance (IBD) in badger population

Using the package PopGenReport [43] in R [44], we constructed a microsatellite distance matrix (Smouse and Peakall method) for all unique badgers captured in the study zone, and for the subset of badgers that produced *M. bovis* cultures from the endemic lineage (see below), to ensure we had sufficient power to detect IBD in sub-populations. For both data sets, we then constructed inter-animal Euclidean distance matrices using the R package Geosphere [45]. We then performed a Mantel test, with 10 000 repetitions for IBD using the package ade4 [46] in R. For the larger meta-population, Mantel tests and linear regressions were carried out for each capture year, with an analysis of covariance carried out to compare the slopes of each genetic distance vs Euclidean distance relationship.

### Preliminary phylogenetic analyses

The most appropriate nucleotide substitution model for our phylogenetic analyses of the FASTA alignments of informative SNPs was assessed using the modelTest function of the package Phangorn [47] in R. Specifically, the fit of the general time-reversible (GTR), Jukes–Cantor and Hasegawa–Kishino–Yano (HKY) models to the data was assessed. The nucleotide substitution model with the lowest AIC (GTR – AIC: 26860.39) was used to build a maximum-likelihood phylogeny using RAxML v8 [48]. The autoMRE rapid bootstrapping search method in RAxML was selected and stopped after 4000 replicates. The phylogeny was visualized and assessed in ggtree in R [49]. The presence/absence of a temporal signal in the phylogenetic data of the endemic clade was assessed using the program TempEst v1.5.1 [50]. After selecting the reference strain (AF2122/97) as the best fitting, outgroup root isolate, which also maximizes the temporal signal, the root-to-tip divergence model was fitted using the residual mean squared method.

To test the significance of the temporal signal in our data set, we randomized the tip dates for the 302 chosen isolates from the endemic clade, in 10 replicate analyses, as per Firth *et al.* [51]. Tip dates were randomized using the Tipdatingbeast package [52] in R. The original data set and each replicate were subjected to beast 2 (Bayesian evolutionary analysis by sampling trees) [53], skyline and constant population, and coalescent analyses, using relaxed and strict clock models, the GTR nucleotide substitution model and a chain length of 200 000 000 MCMC steps, of which 10 % was discarded as burn-in. After checking convergence in Tracer 1.7.1 [effective sample size (ESS) >200 for all parameters], the median substitution rate for each replicate was compared to those of the non-randomized data set. In addition, we also compared the substitution rates to those inferred from the structured birth death analyses (see below) to verify that not taking population structure into account did not result in biased clock rate estimates.

### Bayesian phylodynamic analyses

In an initial attempt we used structured coalescent models to analyse this data set [54]. We hypothesize that the structured coalescent may not have been an appropriate model to use for phylodynamic inference owing to an observed increase in pathogen effective population size that coincided with the study area’s increased bTB incidence [54]. It is recognized that significant changes to effective population size and inter-species transition rates through time can result in spurious outputs from structured coalescent models that assume some level of homogeneity in both parameters [55].

To determine the transmission dynamics of *M. bovis* in the TVR zone, existing spoligotyping and MLVA data were used to rationally target the historically endemic strain family in this region [7, 9]. Specifically, we applied a structured birth death model using the BDMM package [56, 57] in beast 2 [53]. BDMM is based on the birth death skyline model, which can infer epidemiological parameters from sequence data and account for changes in effective population size [56, 57].

Our study population for estimation of transmission rates was a subset of 302 isolates from the endemic lineage defined below. We randomly selected 248 cattle isolates (1 isolate per annum per herd) and 54 isolates from all unique badgers. All 302 isolates were collected in the period 1986–2017. We implemented BDMM to determine inter-species transition rates using strict and uncorrelated log normally distributed relaxed clock models. We selected the following starting priors: *R*_0_ 1.2 with a log normal distribution, in line with prior estimates for the bTB episystem (see below); time to become uninfectious/detection and removal of 1 year, again with a log normal distribution in line with previously described diagnostic test performance statistics and possibility of *M. bovis* latent infection (see below); a substitution rate of 8×10^−8^ substitutions per site per year (0.34 substitutions per genome per year), again with a log normal distribution, in line with previous estimates [16–22]. For all other parameters we used the default settings. Each BDMM analysis was attempted with three replicates with a chain length of 500 000 000 MCMC steps. Following the removal of a 10 % burn-in, chains were combined using LogCombiner v2.6 [53] and analyses were compared based upon the log likelihood scores, model convergence and posterior support of parameters in Tracer v1.7.1 [58]. Maximum clade credibility (MCC) trees were constructed for combined chains using TreeAnnotator v2.6 [53] using the median ancestor heights criterion.

### Bayesian phylogenetic model selection

For the skyline and constant population models with strict and relaxed clocks, we computed the log marginal likelihood (ML) for all beast models using nested sampling in the NS 1.1.0 package [59] and used this to compute the Bayes factor (BF) between pairs of models where BF=log(ML2)−log(ML1). We used established guidelines on BF support [60] to ascertain best fitting models.

### TransPhylo analyses

We used the best-fitting time-stamped, unstructured strict and relaxed clock phylogenies described above to investigate transmission dynamics further using the reversible-jump MCMC algorithm TransPhylo [61], implemented in the R package of the same name.

Within TransPhylo, to model the generation time (time interval between infection and transmission), we used a gamma distribution with w.shape=1.3 and w.scale=3.33 (mean interval=4.33 years, standard deviation=3.8 years). Median length of time for an infected bovine to become infectious has previously been estimated to be around 100 days [62], but it is recognized that *M. bovis* incubation time can be highly variable, with the possibility of latent infection over many years [63, 64]. Similar assumptions have been made by van Tonder *et al*. [22] in their phylodynamic investigation of the randomized badger culling trial data set, and by Didelot *et al*. [61] in their investigation of a *M. tuberculosis* outbreak.

To model the time between infection and detection (sampling interval), TransPhylo uses another gamma distribution, which by default has the same values as those set for the generation time. However, we wanted to specify parameters that were in keeping with the known epidemiological properties of the bTB epi-system in Northern Ireland. All bovine animals are tested using the SICCT, which has been noted as having a sensitivity of between 52 and 100 %, with median values of 80 and 93.5 % depending on interpretation [65], although some approaches have suggested that SICCT sensitivity may be as low as ~50 % [66]. Regardless of the ‘true’ figure, a substantial proportion of TB-infected cattle tested are misdiagnosed as false negatives, and are only detected in subsequent SICCT tests. It is noteworthy that the diagnostic test used for badgers has similar issues with its sensitivity [30], and given the TVR trapping and testing annual protocol, similar issues with identifying positive cattle have likely also affected this species. Alongside issues of diagnostic sensitivity is the time taken for an infected animal to become detectable via the cell-mediated immune response that is the basis of the SICCT. The latter seems to be independent of infectious dose [67] and can be detectable as early as 3–5 weeks post-infection [65]. For these reasons, we assessed that in both host species there was likely to be considerable uncertainty in sampling interval for both host species. We therefore used a gamma distribution with parameters ws.shape=1.05 and ws.scale=2.85 (mean=3 years, standard deviation=2.8 years).

For both the strict and relaxed clock MCC skyline trees, we ran three separate chains with 2×10^6^ MCMC iterations each, assessing post-run convergence and ESS numbers >200 for all model parameters. From the posterior tree space generated by TransPhylo, we computed a medoid transmission tree from which we determined which pairs of sampled isolates exhibited posterior transmission probabilities of >50 % [61].

### Assessing effect of badger population structure on *M. bovis* spatial partitioning

We sought to determine whether *M. bovis* inter-isolate SNP distance is associated with pairwise Euclidean distance between trapped badger locations (2014–2017), pairwise difference in time of *M. bovis* isolation and pairwise host genetic distance. For *M. bovis* genome sequences derived from badgers infected with the endemic lineage, we constructed two inter-isolate distance matrices: (i) SNP distance using the R package ape [68] and (ii) time of isolation difference using the dist function in R.

Using these *M. bovis* distance matrices and the two already produced to assess IBD in the infected badgers (see above), we performed a multiple regression on distance matrices (MRM) analysis using the R package ecodist [69] with 10 000 repetitions.

## Results

### Sampling of cattle and badgers

A total of 642 *M *. *bovis* isolates were used in this study, of which 611 were collected from badgers and cattle in the TVR zone, and 31 from a neighbouring region. Of the 642 isolates, 15 were sequencing QA duplicate controls as discussed above. Of the 642 survey isolates, 399 (282 cattle; 117 badger) were sampled contemporaneously in the TVR zone during the project (2014–2017). A further 242 historical isolates (232 cattle, comprising 201 from within the TVR zone and 31 from a neighbouring area, and 10 badgers from within the zone) from 1986 to 2013 were available from archived cultures from the zone and its immediate neighbouring regions. Cattle isolates across all years were single isolates per animal from 185 herds. Multiple isolates (*n*=86) were cultured from 24 individual badgers, with single isolates derived from a further 36 badgers. In total, between 1986 and 2017, we collected *M. bovis* isolates from 60 unique badgers and 483 unique cattle (Fig. 1b). Full details of sample locations, year of isolation and species of origin are given in Data S1.

### Spoligotyping and MLVA analysis

Twenty-two MLVA types and 6 spoligotypes were observed in the 642 isolates. From prior analyses [7], the spoligotype and MLVA genotypes could be grouped into eight related ‘strain families’. Each is dominated by a probable founder genotype (source of the family name) with related daughter strains varying by spoligotype and/or MLVA polymorphism. Numbers of isolates per strain family are shown in Table 1a. The MLVA 6, spoligotype 263 family (6.263) was considered to be endemic in the region and accounted for most of the observed isolates [7, 9]. The remaining seven strain families (1.140, 2.142, 3.140, 4.140, 5.140, 19.140 and 20.131) were not likely to be endemic in the TVR cull zone, as each has a home range elsewhere in Northern Ireland [7, 8]. Of the 36 isolates from strain family 20.131, 32 were collected from a region neighbouring the TVR zone and 4 badger isolates were found within the zone. Full details of isolate MLVA genotypes and spoligotypes are supplied in Data S1.

**Table 1.** (a) Number of isolates per strain family sampled in the study area with breakdown of number of isolates per host species. (b) Number of SNPs detected in strain family clades. (c) SNP distances between all pairs of isolates within all eight major lineages found in the TVR zone and associated summary statistics

	MLVA strain families	1.140	2.142	3.140	4.140	5.140	6.263	19.140	20.131
**(a)**	**No. of isolates**	33	4	10	38	2	478	2	36
	**No. of cattle isolates**	33	4	10	33	2	367	2	32
	**No. of badger isolates**	0	0	0	5	0	111	0	4
**(b)**	**No. of SNPs in clade**	186	38	84	92	79	377	13	53
**(c)**	**Min**	0.0	8.0	8.0	0.0	na	0.0	na	0.0
	**First quartile**	10.0	2.0	16.0	8.0	na	5.0	na	6.0
	**Median**	15.0	20.5	21.0	12.0	na	7.0	na	8.0
	**Mean**	17.6	19.5	21.6	11.7	na	7.6	na	8.0
	**Third quartile**	19.0	21.0	27.0	15.0	na	10.0	na	11.0
	**Max**	96.0	27.0	34.0	28.0	79.0	25.0	13.0	18.0
	**St. dev**	20.0	6.2	7.3	6.3	na	4.0	na	3.8

### Sequencing, bioinformatic analyses

The RedDog pipeline was used to process the isolates. Twenty-four (22 cattle and 2 badgers) failed the sequencing QA filters (98 % coverage filter for the reference genome) and were excluded. The remaining 618 survey isolates plus the AF2122/97 control passed all QA filters. Detailed QA meta-data for all 619 isolates are given in Data S2. Forward and reverse reads for all unique (excluding 15 QA repeats and repeat sequencing of AF2122/97 – *n*=603) QA passing isolates are deposited at the National Center for Biotechnology Information (NCBI) Sequence Read Archive (BioProject PRJNA925930). Individual biosample accession numbers for the 603 isolates can be found in Data S1.

From the 619 isolates with good quality sequence reads, 1562 SNPs passed QA calling rules and were used to conduct phylogenetic analyses. Details of all SNPs passing QA, and their location in the reference sequence are given in Data S3.

The 15 randomly selected isolates selected for resequencing for quality assurance purposes exhibited zero SNP distances from their initial sequencing.

In our experience, a minority of slow-growing cultures of *M. bovis* can acquire growth of faster growing contaminant micro-organisms, hence we set the mapping filter to permit some contamination. In our experience, the contamination that arises for a minority of samples (7 isolates of the 302 that went forward to the phylodynamics analysis) does not affect RedDog’s SNP calling. Our QA resequencing data provide empirical evidence of this. Three of these repeat sequences had low percentages of read mapping to the reference sequence (76.1–88.1 %), but all three exhibited zero SNP distances from their initially sequenced duplicates, which all had higher mapping read percentages (98.8–99.8 %).

### Badger microsatellite genotyping and IBD analyses

Seven hundred and sixty-nine unique badgers were captured, location recorded, sampled and successfully genotyped between 2014 and 2018. Microsatellite profiles, capture locations and date of capture can be found in Data S4. Summary population genetic statistics for all animals, per year, are collated in Table S1.

Samples from 45 badgers produced positive *M. bovis* cultures. Spoligotyping and MLVA placed them in the major endemic lineage in the study zone. Capture locations for the 45 endemic strain-positive badgers are illustrated in Fig. S1a.

Across all years, the badger population exhibited consistent levels of IBD, as indicated by significant Mantel tests (*r*=0.11–0.17, *P*<0.05) (Table 2). The slopes of the relationships between genetic distance and Euclidean distance were very similar. Small, significant differences were observed in the ANCOVA (Fig. S2 and Table 2), mainly due to the increased slope of the IBD relationships in 2015, 2016 and 2018, consistent with badger genetic differentiation being observed over shorter distances.

**Table 2.** Badger meta population isolation by distance (IBD) relationship for all sampling years

Cohort genotyped	No. of animals	Mantel test Pearson coefficient *r*	Mantel *P* value	Linear model beta	Linear model *P* value	*R* ^**2**^	One unit differentiation per *x* km distance
**2014**	273	0.11	0.0001	1.6×10^−4^	<2×10^−16^	0.012	6.25 km
**2015**	152	0.16	0.0001	2.1×10^−4^	<2x10^−16*^	0.024	4.74 km
**2016**	97	0.17	0.0001	2.6×10^−4^	<2x10^−16^†	0.030	3.84 km
**2017**	113	0.11	0.0001	1.5×10^−4^	<2×10^−16^	0.011	6.67 km
**2018**	134	0.13	0.0004	1.7×10^−4^	<2x10^−16^‡	0.017	5.88 km
**TB+ve**	45	0.16	0.0024	3.1×10^−4^	1.1×10^−7^	0.030	3.22 km

*2015 significantly different from slopes for 2014, 2016 and 2017.

†2016 slope significantly different from slopes for 2014 and 2015.

‡2018 slope significantly different from slopes for 2014 and 2017.

The 45 *M *. *bovis* culture-positive badgers also exhibited significant IBD (Table 2), similar to that of the larger study population.

### Preliminary phylogenetic analyses

#### All isolates

**Fig. 2.**
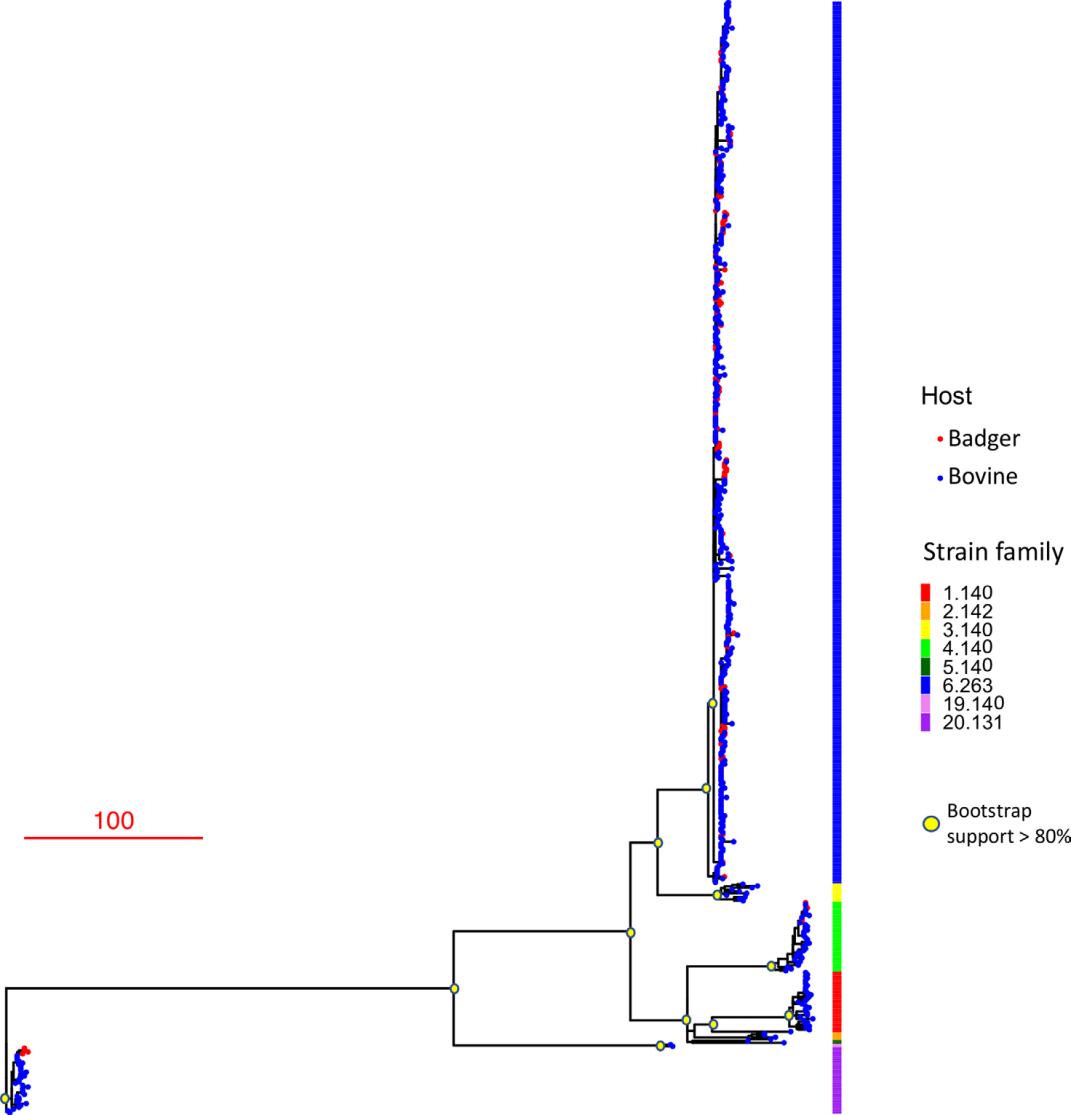
One thousand five hundred and sixty-two SNP maximum-likelihood phylogeny of all 619 isolates that passed sequencing QA. Scale bar represents SNP branch length. Tree is rooted against the outlier 20.131 strain family.

The maximum-likelihood tree constructed in RAxML for all 619 sequenced isolates is shown in Fig. 2. The phylogeny was rooted using the 20.131 strain family as an outgroup, as these isolates are known to derive from an older common ancestor than other extant strains [70]. Eight major lineages, each with high bootstrap support, were observed in the phylogeny. The eight strain families defined previously by MLVA and spoligotyping were monophyletic and in perfect concordance with the SNP-based tree topology. We determined the within-lineage diversity, as defined by total number of SNPs recorded, for each of the eight major lineages observed (Table 1b). Additionally, from distance matrices generated during phylogenetic analyses, pairwise, inter-isolate SNP distance, summary statistics were computed within all eight major lineages (Table 1c). The rest of this paper focuses on the endemic clade. For a wider discussion on non-endemic clades and the general utility of genomic data for *M. bovis* epidemiology and surveillance, please see the Supplementary Material.

#### Endemic lineage – 6.263 strain family

**Fig. 3.**
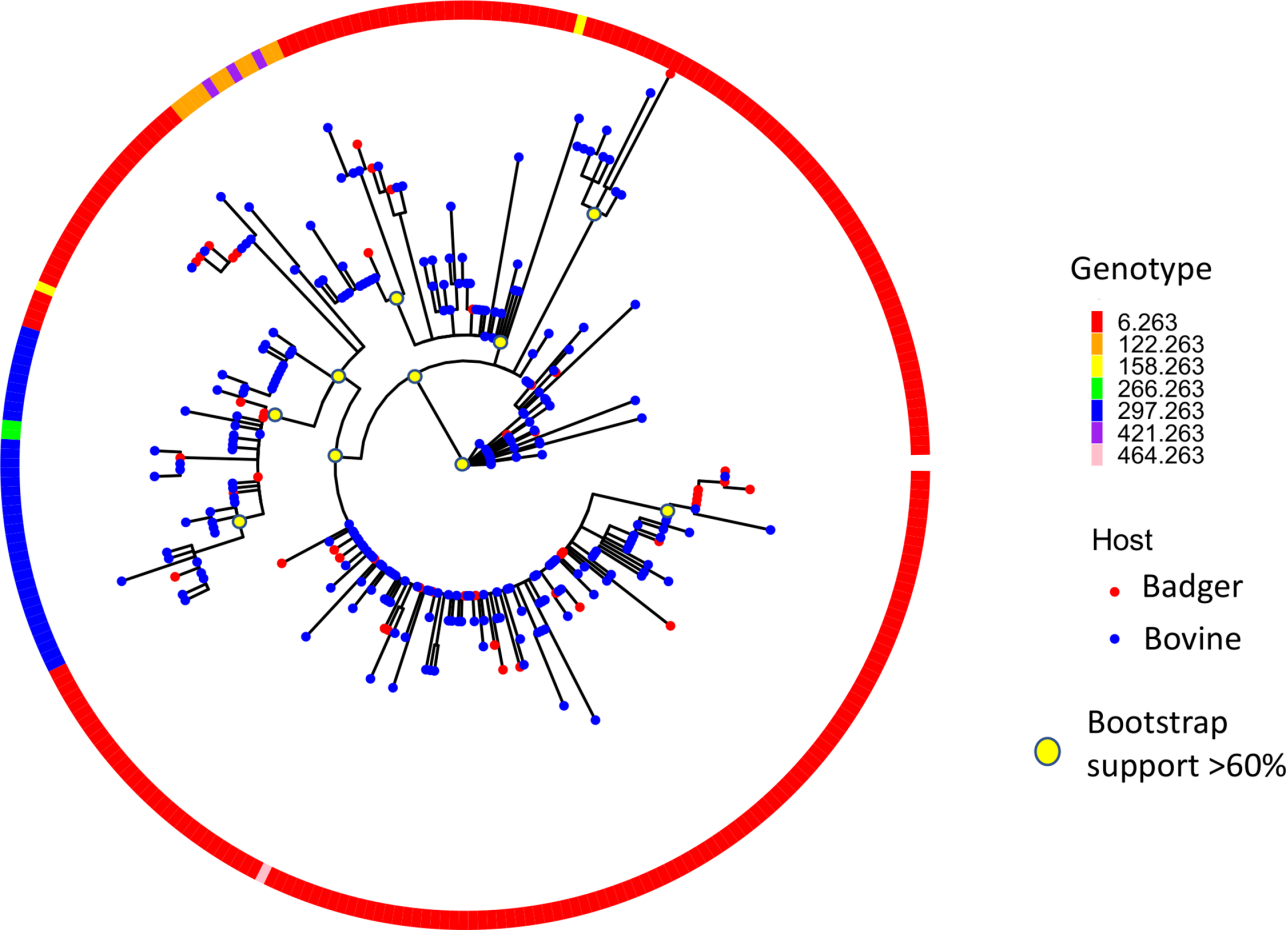
Four hundred and thirty-five SNP maximum-likelihood phylogeny of 302 isolates from the 6.263 endemic lineage in the TVR zone. Tree rooted to the AF2122/97 reference, but reference removed for aid of visualization. Genotype refers to combined spoligotype and MLVA type.

The endemic major lineage of 6.263 presented the best opportunity to investigate *M. bovis* transmission dynamics among cattle and badgers in the TVR zone. 6.263 has been consistently associated with the study area for over two decades in local cattle and badgers, unlike lineages whose home range is elsewhere in Northern Ireland [9]. A higher resolution SNP phylogeny of 6.263 is shown in Fig. 3, using the subset of 302 isolates described above. Cattle and badger isolates were observed in all sub-lineages of the endemic lineage, with no sub-lineages made up of isolates exclusively from a single host species. Major sub-lineages had good bootstrap support (>90). A bar plot showing frequency of isolates per host species taken from the endemic clade over the period 1986–2017 is presented in Fig. S3. A smaller maximum-likelihood phylogeny of the 45 endemic clade badgers is presented in Fig. S1b.

**Fig. 4.**
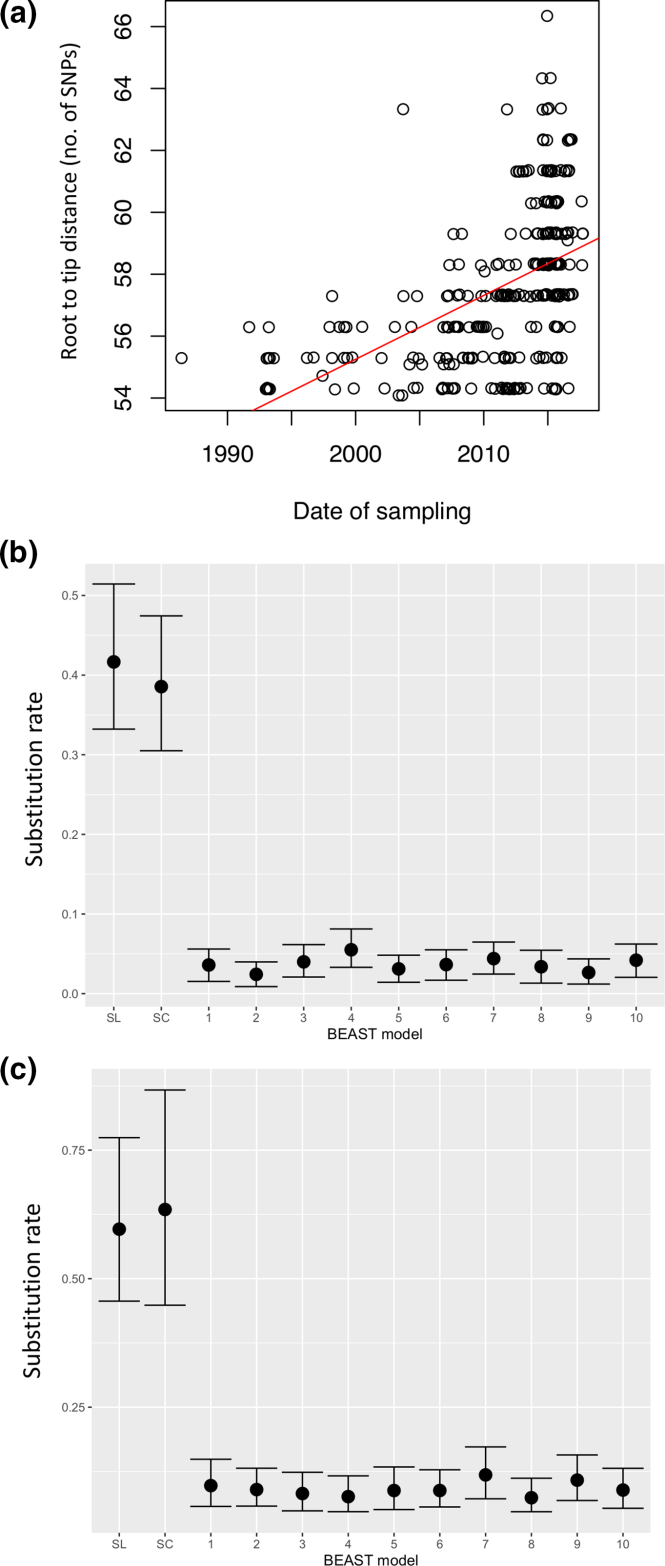
Endemic clade temporal signal tests. (a) Conservative test with Tempest; (b) beast strict clock skyline (SL) and simple constant population size coalescent (SC) models vs 10 randomized tip date data sets; (c) beast relaxed clock skyline (SL) and simple constant population size coalescent (SC) models vs 10 randomized tip date data sets.

The *n*=302 endemic lineage phylogeny analysed in TempEst, rooted against the AF2122/97 reference genome, exhibited a positive correlation between genetic divergence (root to tip distance) and sampling time, with moderate evidence of temporal signal (*R* ^2^=0.25; *P*<0.001), and a conservative clock rate of approximately 0.22 substitutions per genome, per year – Fig. 4a. All 10 tip date randomized replicate data sets run using the constant population size unstructured coalescent and skyline models, with strict (Fig. 4b) and relaxed clocks (Fig. 4c), exhibited similar substitution rates, all of which were considerably lower than the substitution rate inferred from the non-randomized data sets (see below) and exhibited no overlap in 95 % highest posterior density (HPD) interval. This confirmation of the presence of a temporal signal permitted further analyses using Bayesian phylogenetic methods within beast 2 [51].

### Bayesian phylogenetic analyses

#### Unstructured coalescent – constant population size and skyline analyses

**Fig. 5.**
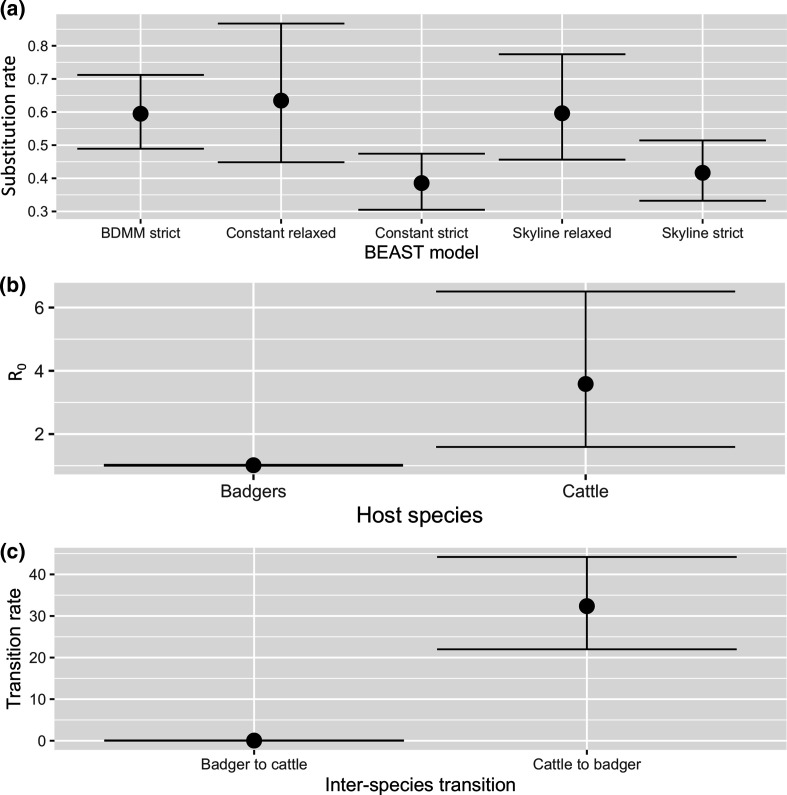
Birth death model parameter outputs. (a) Median substitution rate and 95 % HPD interval for BDMM strict clock vs strict and relaxed clock skyline and simple constant population size coalescent models; (b) BDMM strict clock model median and 95 % HPD interval *R*_0_ estimates; (c) BDMM strict clock model median and 95 % HPD interval interspecies transition rates expressed as transitions per lineage per year.

Unstructured coalescent models converged on similar intra-method values across all parameters. Median substitution rates per genome per year were in keeping with what has been reported before for *M. bovis* (skyline strict clock – 0.42 95 % HPD interval 0.33–0.51; constant population size coalescent strict clock – 0.39 95 % HPD interval 0.31–0.47; skyline relaxed clock – 0.60 95 % HPD interval 0.46–0.77; constant population size coalescent relaxed clock – 0.63 95 % HPD interval 0.45–0.87). All four substitution rates exhibited substantial overlap in their 95 % HPD intervals (Fig. 5a) and suggested that the time to most recent common ancestor (tMRCA) of the endemic clade occurred 32–45 years before the date of the most recent sample in 2017 (Table S2). Both skyline analyses indicated a substantial rise in the effective population size of the endemic clade coincident with the observed increase in disease incidence from 2011 to 2012 (Figs S4 and S5). Nested sampling results indicated that the unstructured analyses with the highest log marginal likelihood were the skyline analyses (Table S3). Only the relaxed skyline Bayes factor had sufficient support to suggest that it was a better fit compared to the relaxed clock constant population size, although we elected to perform TransPhylo analyses on both skyline tree types, as they represented the best approximation of population size dynamics in the endemic lineage, given known changes in disease incidence.

#### Structured birth death model

The MCMC for the relaxed clock BDMM failed to converge after multiple attempts even when using a highly constrained prior distribution of the clock rate. Consequently, we report no findings for this version of the model.

Conversely the three replicate BDMM chains for the strict clock model converged to similar intra-method values across all parameters. The median observed evolutionary rate was 0.59 substitutions per genome per year, with a wide 95 % HPD interval (0.49–0.71) that exhibited overlap with three of the four unstructured coalescent analyses (Fig. 5a). The estimated tMRCA from the model was 31 years before the date of the most recent sample in 2017 (95 % HPD interval 31.0–32.9).

**Fig. 6.**
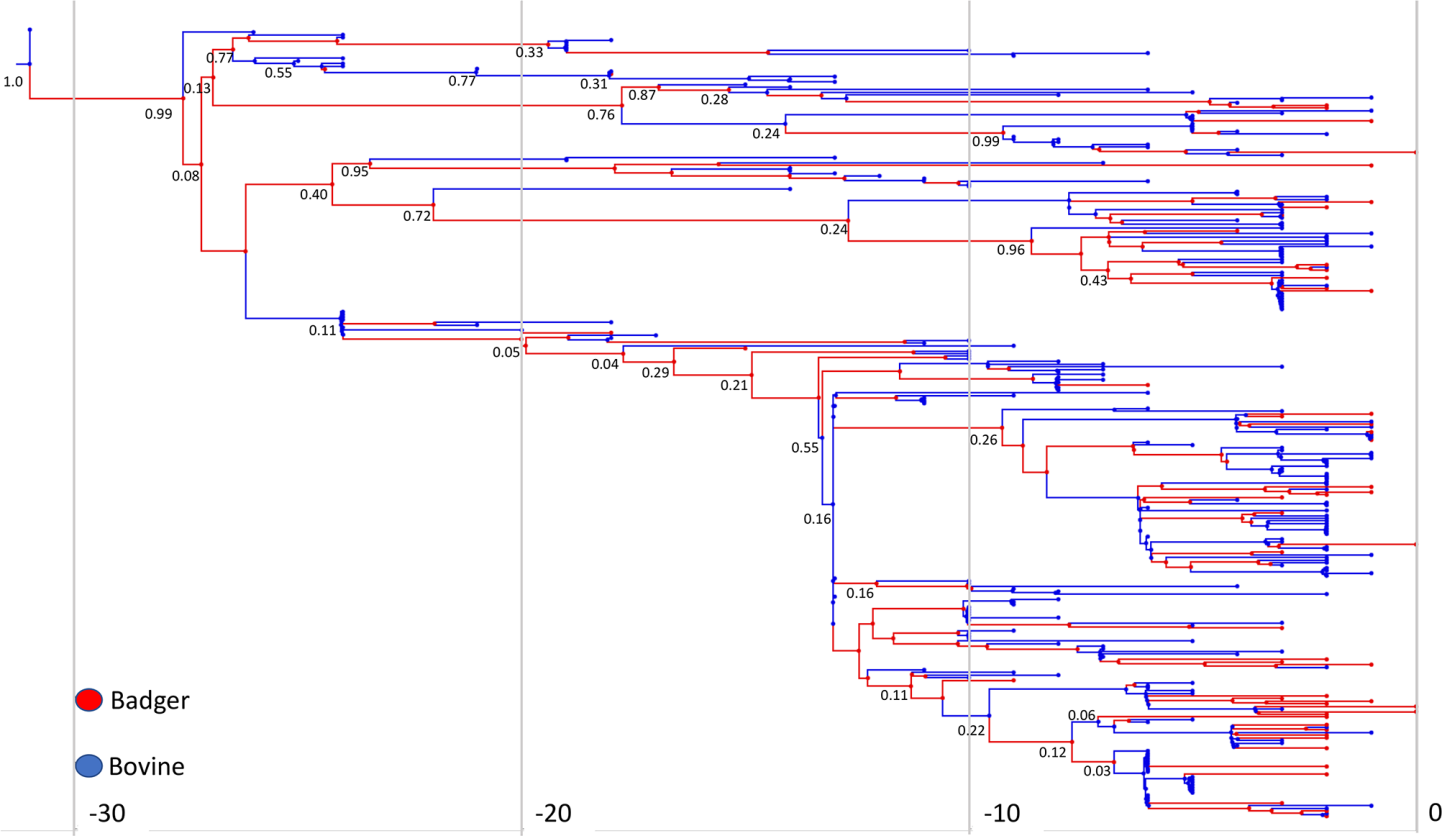
BDMM strict clock maximum-clade credibility transmission tree. Posterior probabilities for major branches/lineages are annotated. Time is displayed on the *x*-axis as number of years before the last sampled isolate from 2017.

A time-stamped, MCC tree from the analysis is shown in Fig. 6, although posterior support for many of the observed clades and major lineages was low. Indeed, while not shown on Fig. 6 for aesthetic reasons, posterior support for the most recently evolved sub-branches was particularly poor.

The model estimated the median time to become uninfectious/detected to be 58 days (2 months), but with a wide 95 % HPD interval of 34–180 days (1–6 months).

Median *R*_0_ for the badgers in this study was estimated to be 1.01 (95 % HPD interval 1.00–1.03), while for the cattle it was estimated to be 3.58 (95 % HPD interval 1.59–6.51) – see Fig. 5b.

Mean badger-to-cattle transition rates were estimated to be 0.04 transitions per lineage per year (95 % HPD interval 0.01–0.08), while cattle-to-badger transitions were estimated to be 32.37 transitions per lineage per year (95 % HPD interval 21.99–44.19) – see Fig. 5c.

#### TransPhylo analyses

The triplicates of the strict and relaxed clock skyline TransPhylo runs exhibited MCMC convergence for all parameters, with ESS of over 200. A summary of the main findings of all six runs are shown in Table 3. *R*_0_ for the endemic clade epi-system was observed to vary between 1.70 and 2.48, and the algorithm estimated that the proportion of the number of truly infected animals (badgers and cattle) sampled was between 0.38 and 0.42.

**Table 3.** TransPhylo outputs for triplicate runs of strict and relaxed clock models

	No. of sequence pairs >50 % transmission posterior prob	No. of unique sequences and % of endemic clade	Reproductive no. *R*_0_ (mean ±)	Proportion of outbreak sampled (mean ±)	No. of bovine-to-bovine transmission pairs (% of total sequence pairs)	No. of badger-to-badger transmission pairs (% of total sequence pairs)	No. of bovine-to-badger transmission pairs (% of total sequence pairs)	No. of badger-to-bovine transmission pairs (% of total sequence pairs)
**Strict clock run 1**	24	45 (14.9 %)	2.06 (1.87–2.26)	0.38 (0.30–0.46)	22 (91.6 %)	0 (0 %)	1 (4.2 %)	1 (4.2 %)
**Strict clock run 2**	29	56 (18.5 %)	1.70 (1.54–1.87)	0.42 (0.33–0.51)	26 (89.7 %)	0 (0 %)	2 (6.9 %)	1 (3.4 %)
**Strict clock run 3**	24	45 (14.9 %)	2.06 (1.87–2.26)	0.38 (0.31–0.45)	22 (91.6 %)	0 (0 %)	1 (4.2 %)	1 (4.2 %)
**Relaxed clock run 1**	37	68 (22.5 %)	2.49 (2.26–2.72)	0.37 (0.30–0.45)	35 (94.6 %)	0 (0 %)	1 (2.7 %)	1 (2.7 %)
**Relaxed clock run 2**	37	68 (22.5 %)	2.48 (2.25–2.73)	0.38 (0.31–0.46)	35 (94.6 %)	0 (0 %)	0 (0 %)	2 (5.4 %)
**Relaxed clock run 3**	34	62 (20.5 %)	2.48 (2.25–2.73)	0.38 (0.31–0.46)	33 (97.0 %)	0 (0 %)	0 (0 %)	1 (3.0 %)

The medoid, relaxed and strict clock transmission trees outputted by TransPhylo (Figs S6 and S7) across all runs indicated that a substantial minority of isolate pairs exhibited posterior transmission probabilities of >50 % – see Table 3. A common feature across all analyses was that most of these pairs (90–97 %) represented bovine-to-bovine transmission events. No pairs representing badger-to-badger transmission were observed. Inter-species transmission pairs were rare, accounting for only 3–10 % of those observed across all replicates, with both cattle-to-badger and badger-to-cattle transmission events inferred (Table 3).

Across all strict and relaxed clock models, the distributions of both time from infection to transmission (Fig. S8a, b) and time from infection to detection (Fig. S9a, b) were positively skewed.

For the strict clock models, time from infection to transmission had a median value of 0.23 years/83 days, with a mean value of 1.18 years/431 days. The data indicated that ~50 % of infected animals (both species) were involved in a transmission event within 1 year of infection. Time from infection to detection had a median value of 0.2 years/73 days and a mean of 1.1 years/402 days. Approximately 50 % of infected animals (both species) were detected within 1 year of infection.

For the relaxed clock models, time from infection to transmission was observed to have the median value of 0.173 years/64 days with a mean value of 1.3 years/450 days. The data indicated that ~57.4 % of infected animals (both species) were involved in a transmission event within 1 year of infection. Time from infection to detection had a median value of 0.14 years/51 days with a mean value of 1 year/370 days. The data indicated that ~56.6 % of infected animals (both species) were detected within 1 year of infection.

### Effect of badger population structure on *M. bovis* spatial partitioning

From the full model in the MRM analysis, modelling *M. bovis* genetic distance (SNP-based) as a function of inter-badger genetic distance, inter-badger Euclidean distance and inter-* M. bovis* time of isolation difference, we observed that only inter-badger Euclidean distance was significantly associated with *M. bovis* genetic distance (*P*=0.04). However, the overall fit of the model was non-significant (F-test *P*>0.05). Badger microsatellite-derived genetic relatedness was therefore not associated with *M. bovis* SNP-derived genetic differentiation. A full summary of MRM findings is presented in Table 4.

**Table 4.** Multiple regression on distance matrices (MRM) analysis of *M. bovis* inter-isolate SNP distance vs pairwise Euclidean distance between positive badgers, pairwise time difference between *M. bovis* isolations and pairwise microsatellite genetic distance between host badgers. *, *P*<0.05

Model	Variable	Slope	*P* value
**SNP dist~Euclidean dist + time of isolation difference+microsat dist**	Euclidean distance	2.83×10^−4^	0.04*
	Time difference	8.81×10^−1^	0.08
	Microsatellite distance	2.92×10^−2^	0.76

Full model *R* ^2^ = 0.04.

*P* value = 0.10.

F = 14.16.

## Discussion

The present study represented a unique opportunity to investigate, in unparalleled resolution, bTB transmission dynamics between cattle and badgers, for an endemic lineage of *M. bovis *, in a well-sampled study area that had experienced an unusually high incidence of disease. The integration of host wildlife genetic data to inform us concerning the partitioning of pathogen genetic diversity within that host is also unique for this epi-system.

We applied Bayesian phylogenetic methods to investigate transmission dynamics in a systematically sampled, multi-host, endemic disease. Our data shed further light on the intra- and inter-species dynamics of *M. bovis* transmission in an endemic area. They are also useful for informing control policies and comparison to epi-systems in different regions. Our analyses suggest that the endemic 6.263 lineage has been present in the TVR zone since the 1970–80s and the rate of molecular evolution of 0.39 to 0.63 substitutions per genome per year is consistent with previous *M. bovis* phylodynamic studies [19–22].

The presence of bacterial isolates from both cattle and badgers throughout all sub-lineages of the endemic 6.263 lineage indicates likely bi-directional transmission between both species. The BDMM and TransPhylo models both identify bi-directional inter-species transmission events but suggest that infection in this region is driven primarily by cattle. The elevated *R*_0_ in cattle and the very discrepant rates of inter-species transmission inferred by the BDMM model, the predominance of cattle-to-cattle transmission events in the TransPhylo data and the absence of an association between badger population structure and how *M. bovis* genetic diversity is partitioned across the landscape all point to this same conclusion.

The BDMM MCC tree’s low posterior support and the considerable uncertainty in estimation of cattle to badger transition rates and cattle *R*_0_ suggest that caution is necessary in interpreting our findings. Such uncertainty in measurement may have arisen due to the reduced genetic diversity of the endemic clade and the presence of multiple polytomies (Table 1 and Fig. 3). Such features are to be expected in any well-sampled outbreak of a slowly evolving pathogen but can cause difficulty in resolving Bayesian tree topologies, affecting the precision of inferences from them [71]. Indeed, it is recognized that low-diversity, highly clonal pathogens such as the tuberculosis-causing bacilli can be very challenging to apply Bayesian phylodynamic methods to [61]. Despite this caveat, we believe the totality of the evidence we describe above supports the general inferences. It is unfortunate that the relaxed clock BDMM model would not converge, as relaxed clock rates have been shown to describe the evolution of *M. bovis* across lineages well in past studies [17, 19, 20]. However, this is likely a feature of the highly clonal, low-diversity *M. bovis* structure in an outbreak setting, making tree inference with a relaxed clock particularly difficult. Perhaps a mitigating circumstance is that here, the focus is on a single endemic clade/lineage with limited genetic diversity for which a strict clock may be more appropriate. Regardless, for such slowly evolving, clonal pathogens using more than one phylodynamic method may be advisable to gain a better appreciation of the likely transmission dynamics. For this reason, alongside the BDMM model, we also used TransPhylo.

TransPhylo’s inference that only ~40 % of the epidemic in the TVR zone had been sampled is consistent with the fact that much of the bovine TB burden is occulted due to known issues with diagnostic test sensitivity and the fact that not all test-positive cattle and badgers (40–50 %) will produce viable cultures that can be genome sequenced. The inferred distribution of time from infection to detection was also consistent with prior knowledge, with a wide interval and considerable uncertainty. Approximately 50 % of infected cattle being detected within 1 year of being infected is in keeping with the annual testing regimen in Northern Ireland and the known sensitivity of the tuberculin test (see above). Similarly, the inferred distribution of time from infection to transmission is consistent with prior estimates [62] and the possibility of latent infection being a feature of disease as with human tuberculosis [64]. Similar overall sampling representativeness and epidemiological statistics have been observed by van Tonder *et al*. [22]. The BDMM inference of time to become uninfectious ranges from 1 to 6 months, which is broadly in keeping with the positively skewed range of TransPhylo’s time from infection to detection suggesting that ~50 % of infected animals (both species) are detected within 1 year.

The BDMM inferred cattle median *R*_0_ of 3.58 (95 % HPD interval 1.59–6.51) and TransPhylo’s whole clade *R*_0_ of 1.70–2.48 for both hosts are at odds with previously assessed estimates for the general badger and cattle epi-system. Previously this has been assessed to range between 1.03 and 1.19 [72] in Britain. We hypothesize that the elevated *R*_0_ statistics inferred here may be due to the sudden increase in disease incidence in the study zone as described previously, and that this rise may have been driven by increased cattle-to-cattle transmission.

The primarily cattle-driven nature of the epidemic in the region, and the comparatively lower impact of inter-species transmission, was again consistent with the findings of Crispell *et al*., van Tonder *et al*. and Rossi *et al*. [19, 21, 22] in other regions where intra-host effects are known to predominate. The absence of any strongly supported badger-to-badger transmission events in TransPhylo, along with the observation that badger landscape genetic structure is not associated with pathogen genetic structure, is suggestive that badgers may not be playing a major role in disease transmission events.

Badger genetic population structure, whilst remaining stable over the intervention period, had no association with how *M. bovis* genetic diversity was spatially distributed. It is possible that this lack of association is due to factors other than reduced badger-to-badger intra-species transmission dynamics. The endemic lineage, if it were a relatively recent incursion, may have had little time to establish foci of persistent infection in badgers, and diffuse across the landscape through philopatric contact networks. This lineage has, however, been present in the region for ~30–40 years, providing ample time for establishment to occur. Alternatively, perturbation of the badger population, and associated dispersal arising through the application of culling, even at a small scale [73], may have served to obscure any association between pathogen and host population structures. The relative stability of the IBD relationship we have observed and empirical studies determining selective culling has not resulted in perturbation [74, 75] suggest that this is unlikely. Since IBD remains stable, this suggests that badgers living in close proximity are more likely to be closely genetically related. Therefore, if they are playing a major role in disseminating TB among each other, then closely related badgers could be expected to share more closely related *M *. bovis. But they do not. This observation on its own is perhaps not as compelling as it could be if observed over a longer time span, but when taken together with the transmission dynamics findings, again the totality of the evidence we present supports the hypothesis of a relatively reduced role for badgers in bTB transmission and persistence in this region, compared to cattle.

Our BDMM inter-species transmission data contrast with the findings of Crispell *et al*. [19] from the Woodchester Park region of Gloucestershire that found badger-to-cattle transitions were much more common than cattle-to-badgers. We find the opposite, albeit with caveats about the precision of estimates as described above. Without employing detailed, comparative methods, it is difficult to definitively understand why transmission dynamics between the two regions are so divergent. However, it may be due to differences in host density. The Woodchester Park badger population is one of the densest in Europe, with an average of 30–40 badgers km^−2^ during the period covered by the study [19, 70]. The TVR region’s badger population is approximately 8–10 times less dense than this – 3.88 badgers km^−2^ from a County Down-wide survey [28], and ~5.6 badgers km^−2^ as assessed in the TVR study [27]. Conversely, the cattle density in the immediate vicinity of Woodchester Park, in Gloucestershire, is quite variable, with estimates ranging from 25 to 100 cattle km^−2^ [76]. Northern Ireland is recognized as having some of the highest cattle densities in western Europe, with recent estimates suggesting an average of 112 cattle km^−2^ [1]. Host density is a major driver of *M. bovis* persistence [1] and it is conceivable that the relative densities of cattle and badgers in different regions, alongside other factors, may affect transmission dynamics. The observed disparity between epi-systems suggests that there may be no simple bTB transmission paradigm on which to base all interventions. Such heterogeneity in regional disease epidemiology, both at temporal and spatial scales, may well call for a more heterogeneous approach in the application of disease eradication schemes.

An important consideration from this study is that while our data are supportive of badgers playing a lesser role in intra-species bTB transmission dynamics in this region, this may not be capturing the full impact of badger-to-cattle transmission, which seeds new infection into herds. Subsequent within-herd ‘amplification’ by cattle-to-cattle transmission may mean the initial seeding event has greater impact than that standalone event, resulting in an outsized contribution to disease spread, as has been postulated before from the UK randomized badger culling trial (RBCT) data. While the badger-to-cattle contribution was estimated at 5.7%, this was modelled to amplify to ~52 % (bootstrap 95 % CI: 9.1–100 %), although the confidence intervals were very wide [77]. Owing to the low rate of molecular evolution observed in *M. bovis *, the phylogenetic methods we employ lack the resolution over shorter outbreak time scales to identify such amplification events and inform on their impact. Indeed, this latter point is an even more broadly salient one – it is recognized that it is difficult to elucidate transmission dynamics for monomorphic pathogens with low rates of evolution using genomic data alone [5], as has been noted before for another member of the MTBC, *M. tuberculosis* [61, 78].

## Study limitations

A separate issue with our findings is that they come from a badger population undergoing selective culling and vaccination, both interventions that are likely to affect interspecies disease transmission dynamics [79, 80]. However, any study seeking to harvest systematically sampled, culturable *M. bovis* from wildlife would have involved disturbance and culling of badgers for post-mortem and pathogen isolation. The application of vaccination is admittedly a different matter, however, and without the necessary non-vaccinated control population in which to study transmission dynamics, we are unable to determine the likely impact of the 5 years of vaccination. The study area itself may be considered ‘unusual’ in that it experienced an incidence of disease unlike that observed in other areas of Northern Ireland, and may only therefore be representative of regions experiencing large outbreaks substantially different from background infection levels.

## Conclusions

We describe how in a small region of Northern Ireland cattle-associated transmission appears to drive bTB disease dynamics. There may, however, be regional heterogeneity in the epidemiology of bTB. Further work in other regions of the UK and Ireland is required to assess just how heterogeneous disease dynamics may be. If substantial heterogeneity is observed, it may be advisable for different regions to adopt bespoke eradication schemes tailored to the prevailing host dynamics in their areas, leading to superior control outcomes.

## Supplementary Data

Supplementary material 1
