# Supplementary material for: Genomic epidemiology of Mycobacterium bovis infection in sympatric badger and cattle populations in Northern Ireland
Source: Microb Genom. 2023 May 25;9(5):mgen001023. doi: 10.1099/mgen.0.001023 (PMC10272874; doi:10.1099/mgen.0.001023)
Supplement: Supplementary material 1 [file mgen-9-1023-s001.pdf]

Supplementary Materials

Genomic epidemiology of *Mycobacterium bovis* infection in sympatric badger and cattle populations in Northern Ireland.

Assel Akhmetova[1], Jimena Guerrero[2], Paul McAdam[3], Liliana C.M. Salvador[4], Joseph Crispell[5], John Lavery[6], Eleanor Presho[7], Rowland R. Kao[8], Roman Biek[1], Fraser Menzies[9], Nigel Trimble[9], Roland Harwood[9], P. Theo Pepler[1], Katarina Oravcova[1], Jordon Graham[10], Robin Skuce[7], Louis du Plessis [11,12], Suzan Thompson[7], Lorraine Wright[7], Andrew W. Byrne [7,13], Adrian R. Allen[7].

1 - University of Glasgow, Glasgow, UK.

2 - Centro de Investigacion en Alimentacion y Desarrollo A.C., Hermosillo, Sonora, Mexico

3 – Fios Genomics, Edinburgh, UK.

4 – Department of Infectious Diseases, College of Veterinary Medicine, University of Georgia, Athens, GA, USA.

5 – Foreign, Commonwealth and Development Office, Glasgow, UK.

6 – Department for the Economy, Belfast, UK.

7 – Agrifood and Biosciences Institute, Belfast, UK.

8 - University of Edinburgh, Roslin Institute, Edinburgh, UK.

9 – Department of Agriculture, Environment and Rural Affairs (DAERA), Belfast, UK.

10 – Farmvet Systems Ltd, Moneymore, UK.

11 – Dept. Biosystems Science and Engineering, ETH Zurich, Basel, Switzerland.

12 – Swiss Institute of Bioinformatics, Lausanne, Switzerland.

13 – Department of Agriculture Food and the Marine (DAFM), Dublin, Ireland.

Supplementary Tables

Year	N	Mean Na	Mean He	Mean Ho	Mean Fis	Mean AR
**2014**	273	4.9	0.52	0.49	0.06	4.77
**2015**	152	5.0	0.51	0.49	0.04	4.81
**2016**	97	4.6	0.52	0.50	0.04	4.53
**2017**	113	5.1	0.52	0.48	0.09	5.05
**2018**	134	4.8	0.51	0.49	0.04	4.73
**ALL YEARS**	769	4.9	0.52	0.49	0.05	4.78

Table S1 – Badger meta population genetic summary statistics averaged across 14 microsatellite loci. N = no. of animals genotyped successfully; Na = no. of alleles observed per locus; He = expected heterozygosity; Ho = observed heterozygosity; Fis = fixation index (level of inbreeding per locus); AR = Allelic richness.

	tMRCA (yrs before 2017)	95% HPD
**Skyline strict clock**	32.4	31.0-36.8
**Skyline relaxed clock**	32.3	31.0-36.8
**Simple coalescent strict clock**	41.9	33.3-52.8
**Simple coalescent relaxed clock**	45.7	32.0-72.3

Table S2 – Endemic clade time to most recent common ancestor (tMRCA) for strict and relaxed clock variants of the skyline and simple constant population coalescent phylogenetic models.

Model	Log Marginal Likelihood (ML)	SD	Bayes factor (BF)	$2\sqrt{SD_2^2 + SD_1^2}$	Model comparison
Strict constant	-7266.61	5.13			Strict skyline
Strict skyline	-7258.94	5.13	7.67	14.51	Strict constant
Relaxed constant	-7745.35	5.1			Relaxed skyline
Relaxed skyline	-7696.13	4.75	49.22	13.94	Relaxed constant

Table S3 – Strict and relaxed clock, skyline and constant population model outputs. Log Marginal Likelihoods and standard deviation (SD). Bayes factor (BF) = logML model 2 – logML model1. $2\sqrt{(SD^2_{model2}+SD^2_{model1})}$ is calculated from the standard deviations of both models being compared.

Provided it exceeds the figure calculated for $2\sqrt{(SD^2_{model2}+SD^2_{model1})}$, BF of 3-20 is positive support for 2nd model being superior & BF of 20-150 is strong support as per Kass and Rafferty, 1995 [60].

Supplementary Figures

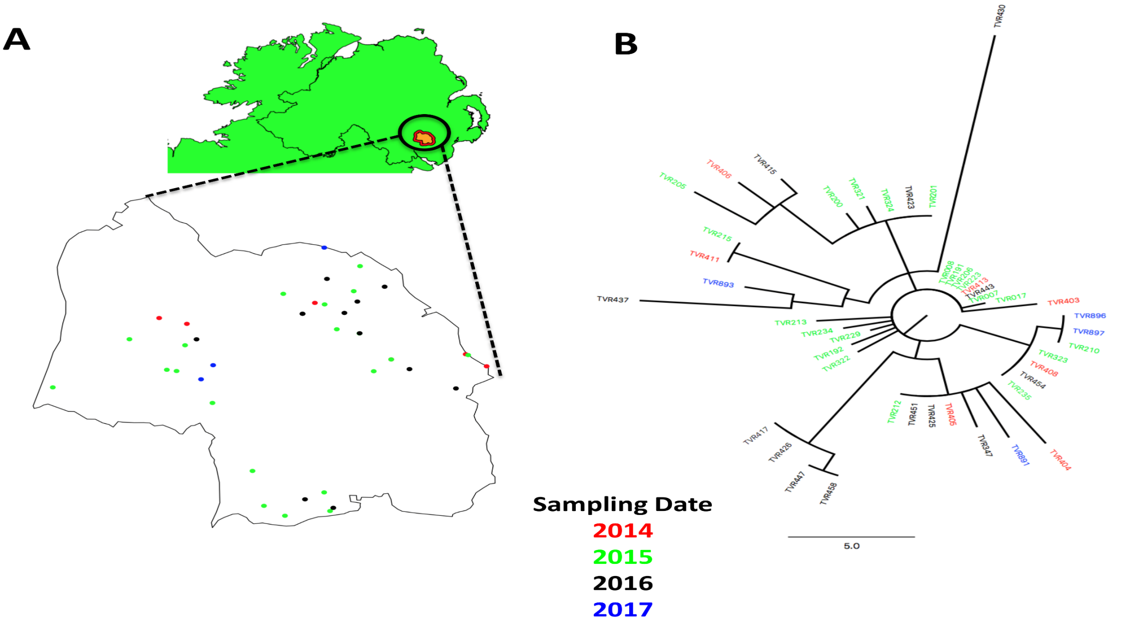

Supplementary Figure S1 – A: Locations of 45 *M. bovis* culture positive badgers by year. B: Maximum likelihood phylogeny of 45 *M. bovis* endemic lineage isolates from TB positive badgers.

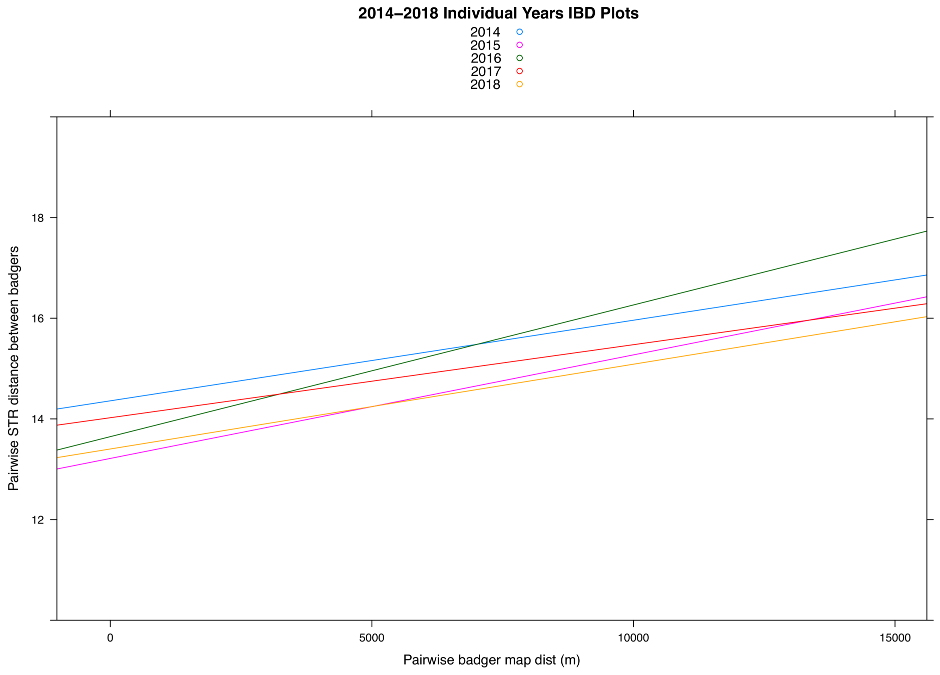

Figure S2 - Linear regressions of badger IBD relationships – pairwise microsatellite / STR genetic distance vs Euclidean distance for all capture years.

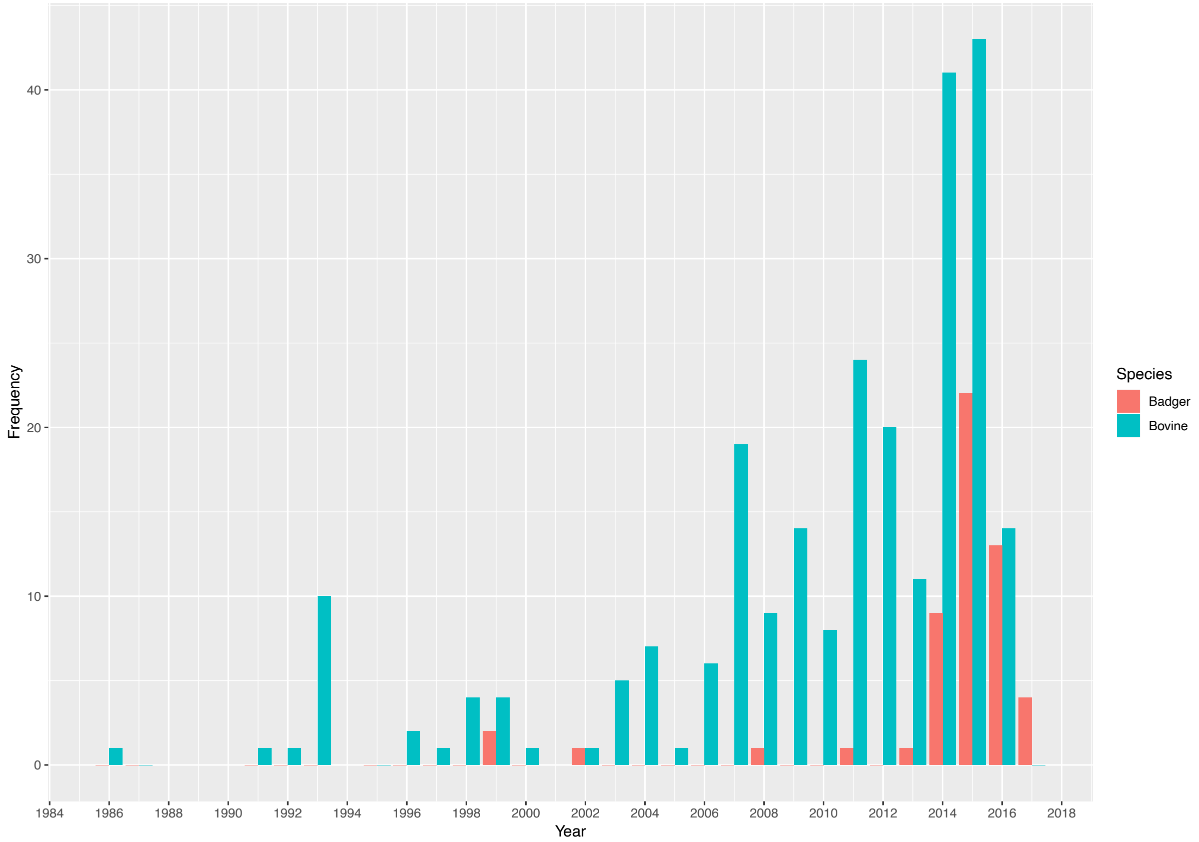

Supplementary Figure S3 – Sampling frequency across all years by host for endemic 6.263 lineage.

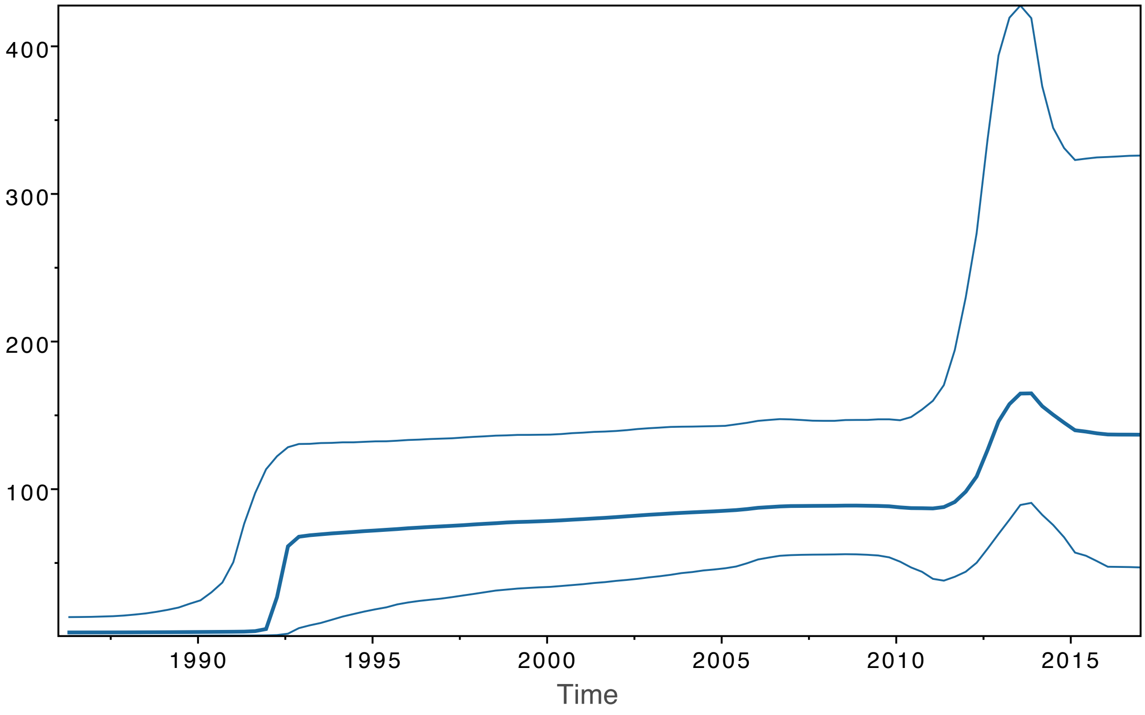

Supplementary Figure S4 - Skyline effective population size of the endemic clade through time plot for the strict clock model.

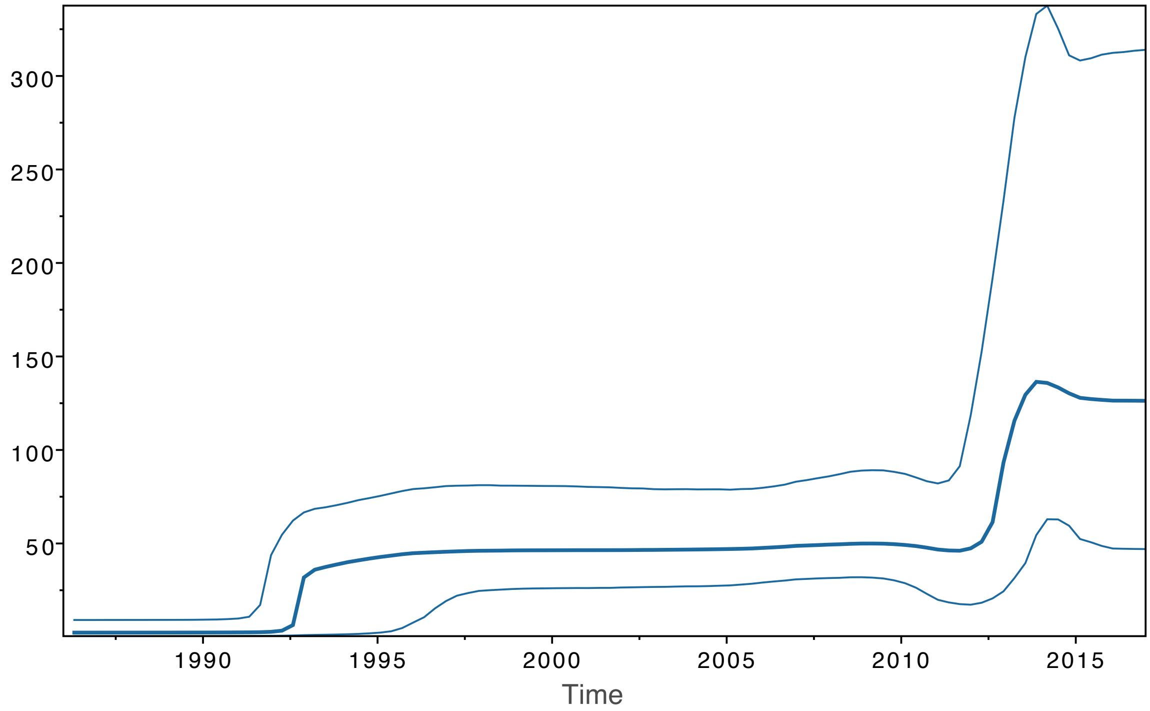

Supplementary Figure S5 – Skyline effective population size of the endemic clade through time plot for the relaxed clock model.

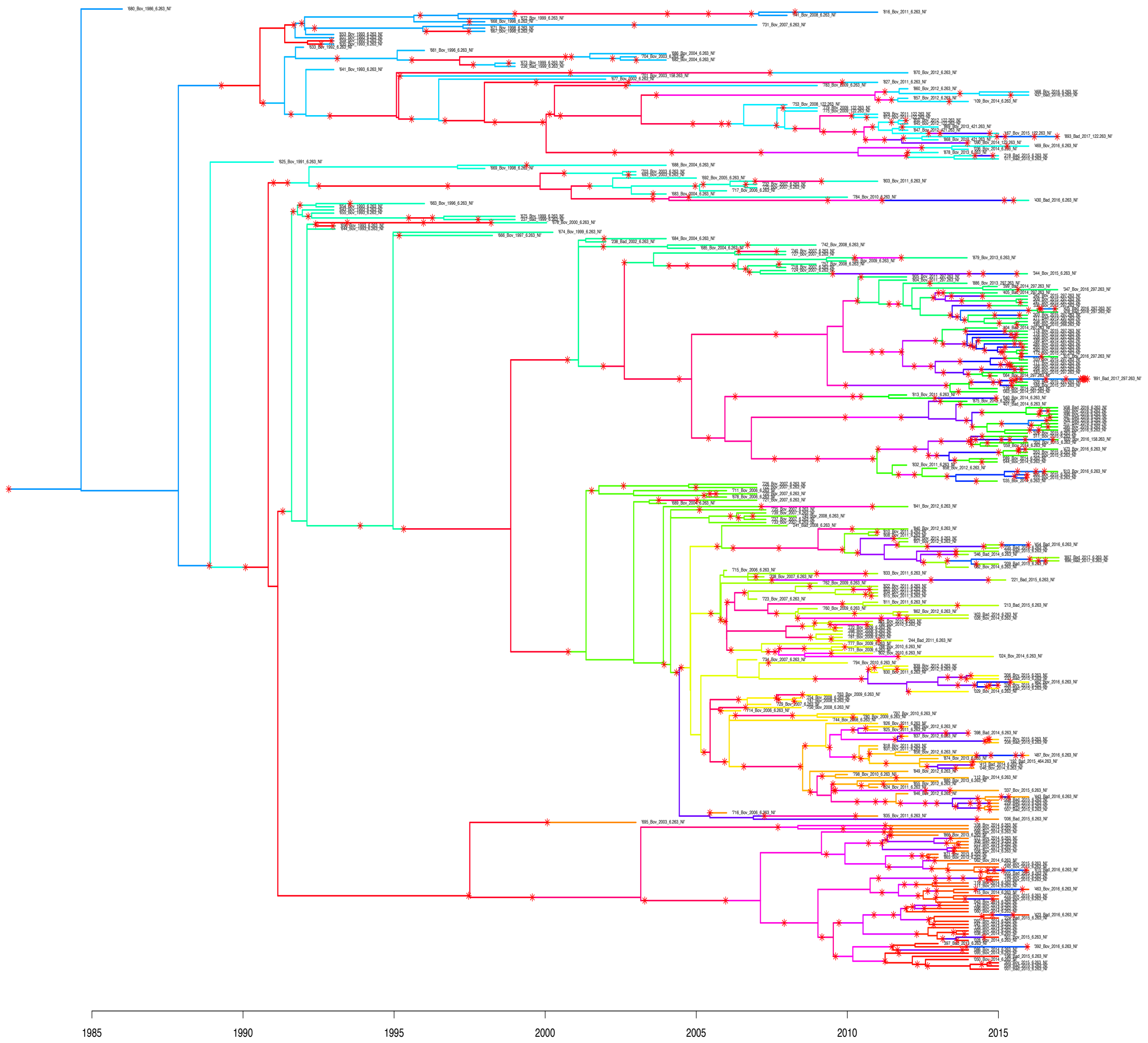

Supplementary Figure S6 – Transphylo medoid transmission tree of the endemic clade for the strict clock model. Branch colour changes and red stars indicate inferred host change events.

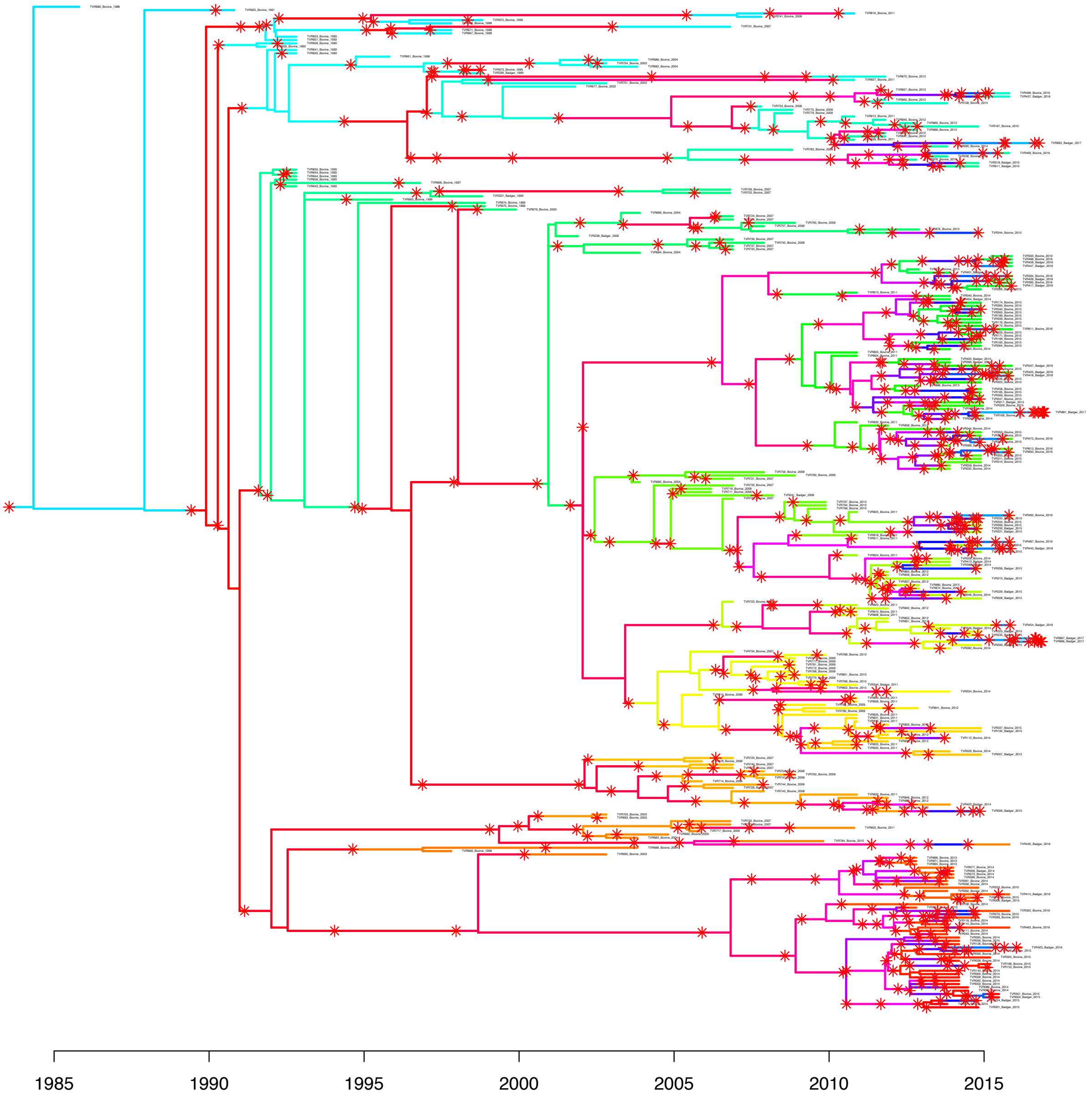

Supplementary Figure S7 – Transphylo medoid transmission tree of the endemic clade for the relaxed clock model. Branch colour changes and red stars indicate inferred host change events.

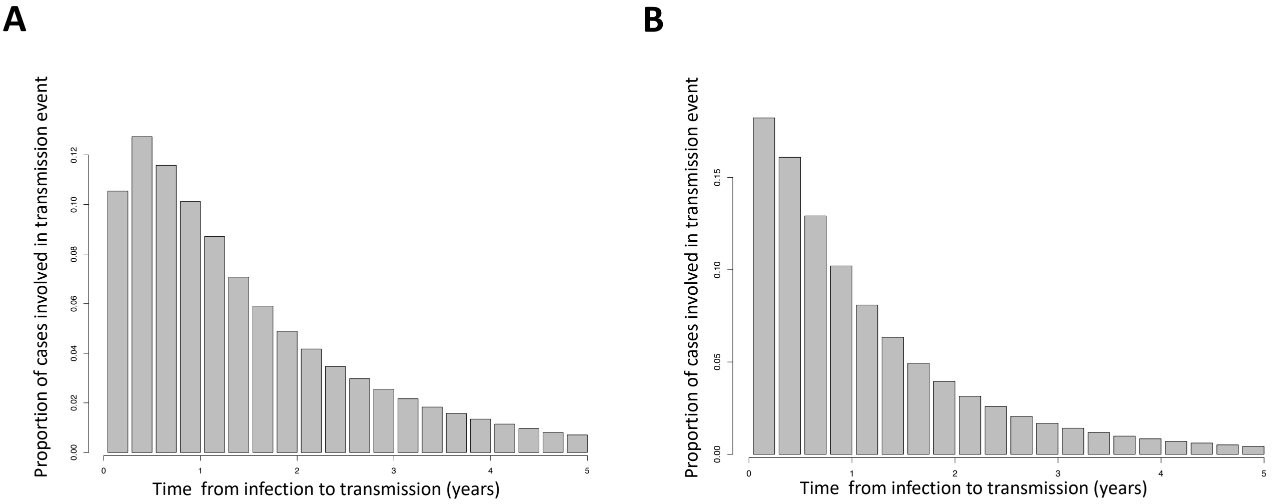

Supplementary Figure S8 – Transphylo distribution of time from infection to transmission (generation time) for **A**: strict clock model, **B:** relaxed clock model.

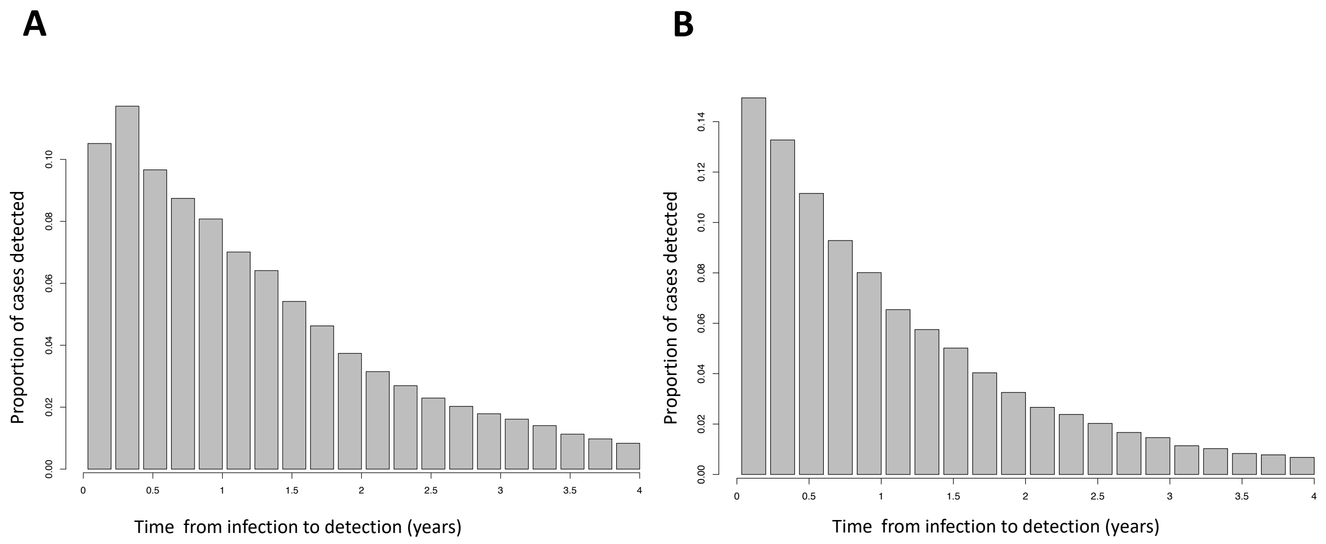

Supplementary Figure S9 – Transphylo distribution of time from infection to detection for **A**: strict clock model, **B:** relaxed clock model.

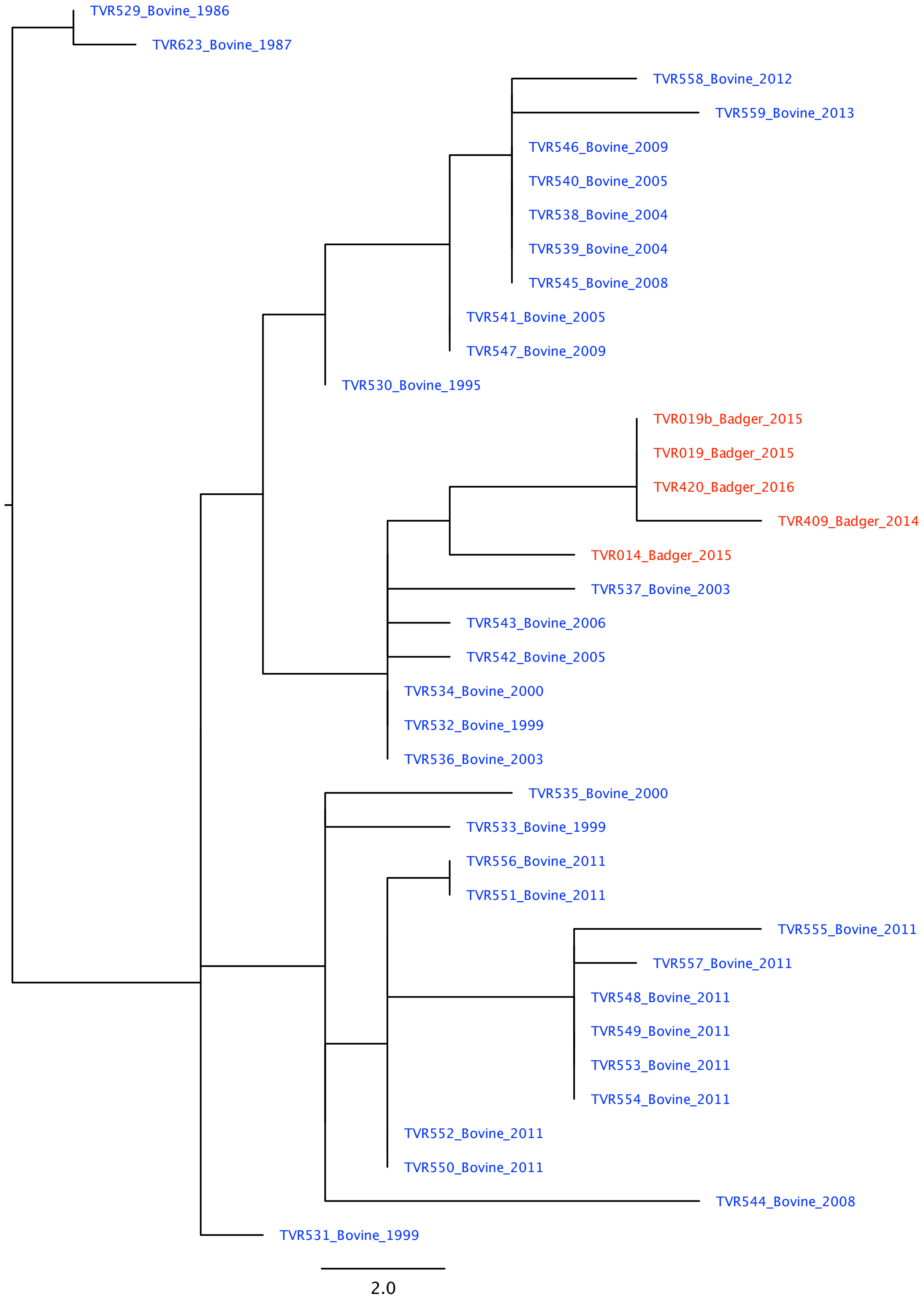

Supplementary Figure S10 - Maximum likelihood phylogeny (53 SNPs) of non-endemic lineage 20.131.

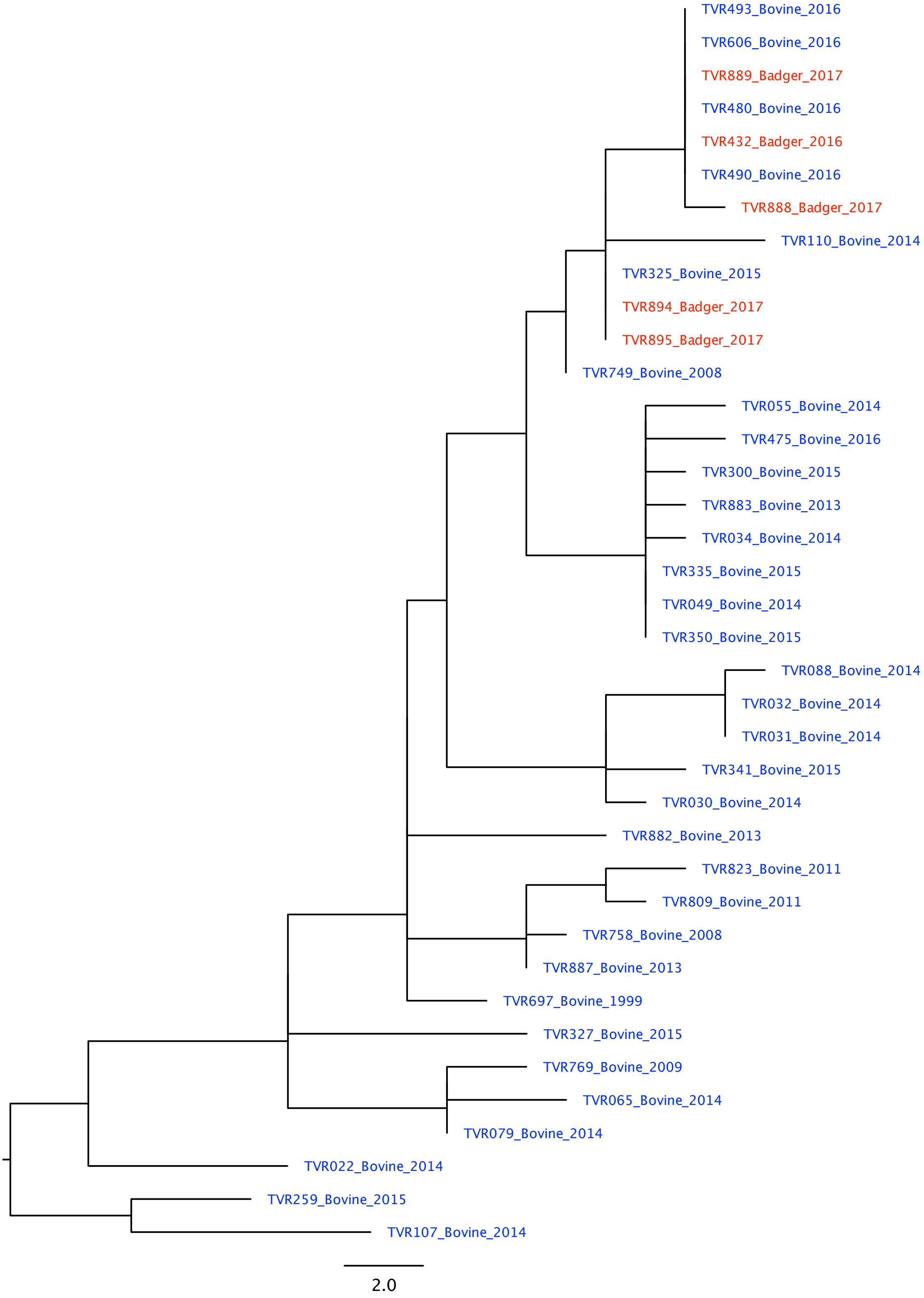

Supplementary Figure S11 – Maximum likelihood phylogeny (92 SNPs) of non-endemic lineage 4.140.

Supplementary Text

Utility of WGS for bovine TB surveillance.

Our data are consistent with the findings that WGS provides unparalleled resolution for epidemiological investigations of zoonotic disease [1] – indexing additional pathogen variation to stratify isolates that are homogeneous according to the classical tuberculosis molecular epidemiological tools of spoligotyping and MLVA [2-3].

Regarding the latter tools, we find perfect congruence between spoligotype and MLVA data and the basal nodes defining major lineages of the phylogeny for all isolates from the TVR region (main text Figure 2). This concordance is a testament to the clonality of *M. bovis* [4-5] and is indicative that existing databases of classical molecular markers, can be used to target 'hotspots' of persistent, endemic infection for closer investigation using WGS.

Contemporary transmission of slowly evolving pathogens, such as members of the *Mycobacterium tuberculosis complex* (MTBC), is typically characterised by little within-clade diversity and resulting reduced SNP distances / phylogenetic branch lengths between isolates [6]. Previous studies have suggested various minimal SNP distance thresholds, for the definition of contemporaneous, epidemiologically linked isolates. Five and twelve SNP distances have been proposed to be consistent with such transmission clusters in *M. tuberculosis* outbreaks in the United Kingdom [7], whilst ten SNP thresholds have been proposed in other studies with *M. tuberculosis* and *M. bovis* [8-12]. Meehan et al (2018) [6] have observed that in *M. tuberculosis*, SNP distances between one and five can represent transmission events up to 10 years apart. Given that MTBC evolution appears to consistently involve relaxed molecular clock like behaviour across lineages [12-14], it is perhaps not surprising that selecting a definitive threshold for contemporary transmission is difficult, and as a result, those set can appear arbitrary. In addition to the issues with relative clock like behaviour of different *M. bovis* lineages, intensity of sample collection and representation of multiple time points can be crucial to establishing robust substitution rates [1]. The general rule of thumb remains however - the shorter the SNP distance between isolates, the more likely they are more closely epidemiologically linked. In this study, the lineage we know to be endemic in the study area from years of MLVA surveillance, exhibits the shortest average pairwise SNP distance between isolates (7.6 s.d. ±4.0 – see Table 1), which is likely indicative of contemporary transmission in the region, and compares favourably to the thresholds discussed above.

The advantage that WGS will provide in disease tracing compared to historical molecular epidemiology methods in the *M. bovis* epi-system, is that outbreaks can be traced back to higher resolution, WGS-defined lineages and sequence types, found in more precise locations than those defined by genetically homogeneous, MLVA home ranges that can cover substantial geographical areas [2-3]. A recent and pertinent example of this, is an outbreak from Cumbria in northwest England, which genome sequencing revealed was linked to the outbreak area under study here [15].

Monitoring of non-endemic lineages

Sequence data are useful for identifying probable incursions of non-endemic disease lineages into new areas, and for potentially determining if the incursion results in contemporary transmission and persistence. Intra-lineage, inter-isolate SNP distances greater than that observed for the endemic 6.263 lineage, are possibly indicative of a lack of contemporary transmission, likely associated with lineages which are non-endemic in the study region as per previous observations from Northern Ireland wide molecular epidemiological surveillance [2-3]. This certainly appears to hold true for lineages 1.140, 2.142 and 3.140, which exhibit average pairwise inter-isolate distances of between 17.6 and 21.6 SNPs. Lineages 5.140 and 19.140 are only represented by two isolates each, but for both lineages, inter-isolate distances are again observed to be larger than the endemic lineage at 79 SNPs and 13 SNPs respectively. Given this observation, and the fact that we know these five lineages are outside of their MLVA defined home ranges [3], it seems probable that they could have arrived in the study area through multiple, long distance, cattle movements. It is noteworthy, but anecdotal, that these lineages are comprised solely of isolates from cattle, suggestive perhaps that the lack of contemporary transmission for these incursive strains has resulted in no infection reaching the wildlife population. However, with deficiencies in sampling, badger 'trappability' and TB test diagnostics as discussed in the supplementary materials, one cannot be definitive that a non-endemic, visiting pathogen lineage has not established a focus of infection in the study area. Only continued surveillance over a wider temporal window could assess that. The establishment of long-term genome-based surveillance systems could in the future help to inform on successful incursions (Gardy and Loman, 2018) [16].

The 20.131 lineage exhibits mean inter-isolate SNP distances which are comparable to that of the endemic 6.263 lineage (main text Table 1). However, only four isolates of this lineage, from two badgers (See Supplementary Figure S10), were found within the study area, with the majority sampled from a neighbouring region in which this strain has a focus of infection. The short inter-isolate distances observed are therefore more likely to be consistent with contemporary transmission in the neighbouring region. It is noteworthy however that the two badgers sampled for this lineage and found in the study zone were found in isolation, with no associated, contemporary, study zone cattle isolates. It could be that badgers have dispersed from the neighbouring region into the study zone, carrying infection with them, which has yet to appear in the cattle population. However, it is also possible that an undetected reservoir of cattle may have entered the study zone and transmitted infection to local badgers. Alternatively, an undetected reservoir not picked up by sampling could be residing within the zone. With so few isolates of the 20.131 lineage from within the study area, and the previously mentioned biases in sampling, it is impossible to be definitive. Again, detailed, longitudinal, genome facilitated surveillance would perhaps be able to inform more fully on this incursive lineage in this region.

Interestingly, one of the historically non-endemic lineages (4.140) [3], does appear to be persisting in the TVR zone, with multiple badger-sourced isolates observed to exhibit shorter inter-isolate SNP distances (0-1) between each-other and cattle from the area (Supplementary Figure S11), consistent with more contemporary transmission events. The latter observation does highlight the usefulness of this WGS based approach for on-going surveillance, and for detecting incursions that establish new foci in new regions. It seems most probable from this case, that new foci of non-endemic lineages are introduced by cattle

movements, with subsequent spill-over to badgers. A similar chain of events has been described for the RBCT area by van Tonder et al (2021) [17].

References

1. Kao RR, Price-Carter M, Robbe-Austerman S. Use of genomics to track bovine tuberculosis transmission. *Rev Sci Tech* 2016;35(1):241-258.
2. Skuce RA, Mallon TR, McCormick C, McBride SH, Clarke G et al. *Mycobacterium bovis* genotypes in Northern Ireland: herd-level surveillance (2003-2008). *Vet Rec* 2010;167(18):684-689
3. Skuce R, Breadon E, Allen A, Milne G, McCormick C et al. Longitudinal dynamics of herd-level *Mycobacterium bovis* MLVA type surveillance in cattle in Northern Ireland 2003-2016. *Infect Genet Evol*: 2020;79:104131.
4. Smith NH, Gordon SV, de la Rua-Domenech R, Clifton-Hadley RS, Hewinson RG. Bottlenecks and broomsticks: the molecular evolution of *Mycobacterium bovis*. *Nat Rev Microbiol* 2006;4(9):670-681.
5. Allen AR, Dale J, McCormick C, Mallon TR, Costello E et al. The phylogeny and population structure of *Mycobacterium bovis* in the British Isles. *Infect Genet Evol* 2013;20:8-15.
6. Meehan CJ, Moris P, Kohl TA, Pečerska J, Akter S et al. The relationship between transmission time and clustering methods in *Mycobacterium tuberculosis* epidemiology. E*BioMedicine* 2018;37:410-416.
7. Walker TM, Ip CL, Harrell RH, Evans JT, Kapatai G et al. Whole-genome sequencing to delineate *Mycobacterium tuberculosis* outbreaks: a retrospective observational study. *Lancet Infect Dis* 2013;13(2):137-146.
8. Bryant JM, Schürch AC, van Deutekom H, Harris SR, de Beer JL et al. Inferring patient to patient transmission of *Mycobacterium tuberculosis* from whole genome sequencing data. *BMC Infect Dis* 2013;13:110.
9. Roetzer A, Diel R, Kohl TA, Rückert C, Nübel U et al. Whole genome sequencing versus traditional genotyping for investigation of a *Mycobacterium tuberculosis* outbreak: a longitudinal molecular epidemiological study. *PLoS Med* 2013;10(2):e1001387.
10. Yang C, Luo T, Shen X, Wu J, Gan M et al. Transmission of multidrug-resistant *Mycobacterium tuberculosis* in Shanghai, China: a retrospective observational study using whole-genome sequencing and epidemiological investigation. *Lancet Infect Dis* 2017;17(3):275-284.
11. Jajou R, de Neeling A, van Hunen R, de Vries G, Schimmel H et al. Epidemiological links between tuberculosis cases identified twice as efficiently by whole genome sequencing than conventional molecular typing: A population-based study. *PLoS One* 2018;13(4):e0195413.

12. Crispell J, Benton CH, Balaz D, De Maio N, Ahkmetova A et al. Combining genomics and epidemiology to analyse bi-directional transmission of *Mycobacterium bovis* in a multi-host system. *Elife* 2019;8.
13. Crispell J, Zadoks RN, Harris SR, Paterson B, Collins DM et al. Using whole genome sequencing to investigate transmission in a multi-host system: bovine tuberculosis in New Zealand. *BMC Genomics* 2017;18(1):180.
14. Menardo F, Duchêne S, Brites D, Gagneux S. The molecular clock of *Mycobacterium tuberculosis*. *PLoS Pathog* 2019;15(9):e1008067.
15. Rossi G, Crispell, J., Brough, T., Lycett, S.J., White, P.C.L., Allen, A., Ellis, R.J., Gordon, S.V., Harwood, R., Palkopoulou, E., Presho, E., Skuce, R., Smith, G.C., Kao, R.R. Phylodynamic analysis of an emergent *Mycobacterium bovis* outbreak in an area with no previous wildlife infections. *J Appl Ecol* 2021;59(1):210-222.
16. Gardy JL, Loman NJ. Towards a genomics-informed, real-time, global pathogen surveillance system. *Nat Rev Genetics* 2018;19(1):9-20.
17. van Tonder AJ, Thornton MJ, Conlan AJK, Jolley KA, Goolding L et al. Inferring *Mycobacterium bovis* transmission between cattle and badgers using isolates from the Randomised Badger Culling Trial. *PLoS Pathog* 2021;17(11):e1010075.
